# Advancements in Fiber-Reinforced Polymer Composites: A Comprehensive Analysis

**DOI:** 10.3390/polym16010002

**Published:** 2023-12-19

**Authors:** Alin Diniță, Razvan George Ripeanu, Costin Nicolae Ilincă, Diana Cursaru, Dănuța Matei, Ramadan Ibrahim Naim, Maria Tănase, Alexandra Ileana Portoacă

**Affiliations:** Mechanical Engineering Department, Petroleum-Gas University of Ploiești, 100680 Ploiesti, Romania; adinita@upg-ploiesti.ro (A.D.); rrapeanu@upg-ploiesti.ro (R.G.R.); icostin@upg-ploiesti.ro (C.N.I.); dianapetre@upg-ploiesti.ro (D.C.); danuta.matei@upg-ploiesti.ro (D.M.); ing_ramadan@yahoo.com (R.I.N.)

**Keywords:** FRP, mechanical properties, manufacturing technologies, applications, environmental conditions, reinforcement

## Abstract

Composites made from fiber-reinforced polymers (FRPs) are a crucial and highly adaptable category of materials widely utilized in numerous fields. Their flexibility and the range of criteria for classification enable the creation of tailored solutions to address distinct requirements in sectors such as civil engineering, aerospace, automotive, and marine, among others. The distinguishing characteristics of FRP composites include the type of reinforcing fiber used, the composition of the matrix material, the employed manufacturing process, the orientation of the fibers, and the specific end-use application. These classification variables offer engineers a versatile structure to determine and select the most appropriate materials and production techniques for their specific needs. Furthermore, the present study aims to reunite the criteria of classification for FRPs and specific manufacturing technologies of FRPs, such as conventional ones (matched die molding, contact molding), automated ones (filament winding, tape lay-up, and fiber placement), and advanced ones (electrospinning and additive manufacturing),with the chronological development of FRPs, insights on material characteristics, and comprehensive design guidelines based on their behavior in different environments of use.

## 1. Introduction

**Figure 1:**
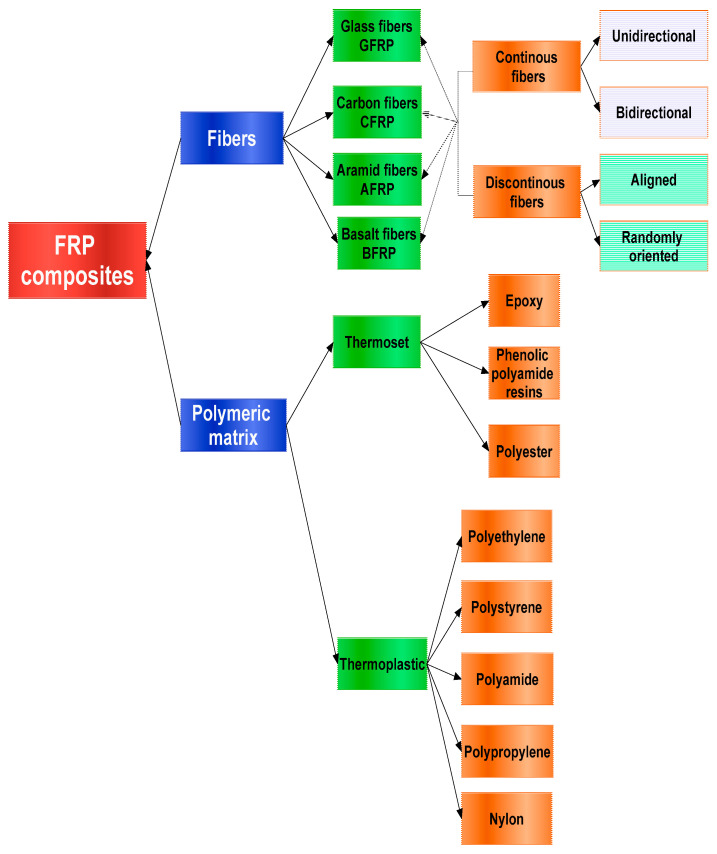
Types of FRP composites.

Fiber-reinforced polymer (FRP) refers to a type of composite material wherein a polymer is strengthened through the incorporation of fibers (Figure 1). These composites belong to a broader category known as composite materials. Such materials are created by blending particles of one or more substances within another material, creating a cohesive network that surrounds and reinforces them.

The composite materials contain two elements, namely the fibers and matrices.

The matrix material in fiber-reinforced polymer (FRP) composites serves as the foundation for the overall performance and characteristics of the material. In thermoset matrices such as epoxy, polyester, and vinyl ester, the focus is on providing structural rigidity and resilience at elevated temperatures. Epoxy, for instance, is known for its high strength and excellent adhesion to fibers, making it suitable for applications where structural integrity is critical, such as in aerospace components. On the other hand, thermoplastic matrices like polyamide and polyethylene offer malleability and reversibility, allowing for reshaping and repairability. This flexibility in design and the ability to withstand impact make thermoplastics advantageous in applications requiring durability and recyclability. The matrix–fiber interaction is crucial, influencing properties like tensile strength, modulus, and flexural strength. The choice of matrix also dictates manufacturing processes, with thermosets like polyester offering cost-effective and faster curing options, while thermoplastics involve more intricate procedures. The adhesion strength between the matrix and fibers, along with the matrix’s role in controlling fiber orientation, contributes to the anisotropic properties of the composite. In essence, the matrix material composition plays a nuanced role in tailoring FRP composites, allowing engineers to adjust characteristics to meet specific needs in diverse industries.

The composite materials contain two elements, namely the fibers and matrices. For FRP composites, the main components are fibers and matrices, but their composition also includes fillers and additives. The fibers, with their high elastic modulus, significantly contribute to the mechanical properties of FRPs. Concurrently, resins play a crucial role in transferring and distributing stresses among fibers, protecting them from both mechanical and environmental damage. Beyond these primary elements, fillers play a role in minimizing shrinkage and overall cost. Additionally, additives are incorporated to enhance the physical and mechanical properties of the composite, improving both its performance and workability.

In construction, three primary fiber types stand out: carbon, glass, and aramid. The acronym name of the composite often reflects the type of reinforcing fiber used; for example, CFRP denotes carbon-fiber-reinforced polymer. The crucial distinctions between these fiber types lie in their stiffness and tensile strain properties. Figure 1 presents a summarized classification of FRP composites.

### 1.1. Criteria of Classification for FRPs

A. Based on the type of reinforcing fiber.

Carbon-fiber-reinforced polymer (CFRP): Uses carbon fibers for reinforcement, demonstrating numerous superior performances, including high strength, lightweight, corrosion resistance, and remarkable fatigue resistance [1].

Glass-fiber-reinforced polymer (GFRP): Uses glass fibers, offering good electrical insulation and corrosion resistance, high strength, flexibility, stiffness, and durability. By using proper orientations and compositions of glass fibers, the desired characteristics and functional properties of GFRP composites can be achieved, making them comparable to steel in terms of stiffness while having a lower relative density than steel. Additionally, GFRPs are known for being the most cost-effective [2].

The glass fibers are subdivided into different sub-categories, as presented in Table 1, according to [3].

Aramid (Kevlar)-fiber-reinforced Polymer (AFRP): Uses aramid fibers, providing a balance of strength, stiffness, and impact resistance. Aramid fibers surpass other synthetic fibers by 5–10% in mechanical properties, making them excellent replacements for metal wires and inorganic fibers. In addition to outperforming steel and glass fibers at equal weights, aramid fibers exhibit remarkable heat and flame resistance even at high temperatures [4]. Despite its positive attributes, aramid fibers have certain disadvantages, such as moisture absorption and relatively low compressive strength, restricting their applications in certain industries [4]. According to [3], there are several types of Kevlar fibers, as seen in Table 2. 

Basalt-fiber-reinforced polymer (BFRP):This type has good corrosion resistance and designability [5]. This alternative material is both cost-effective and has exceptional properties compared to glass fibers. Some of the notable benefits of these composites are their high specific mechano-physico-chemical properties, biodegradability, and non-abrasive characteristics [6].

B. Based on the matrix material.

**Figure 2:**
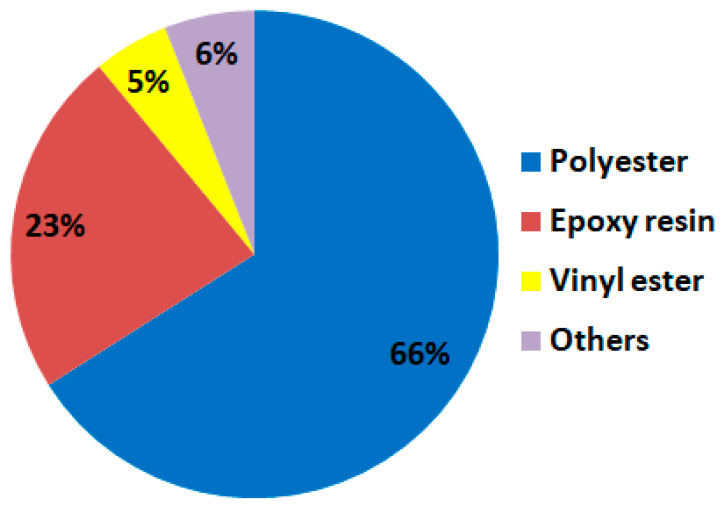
The distribution of thermoset resins in the FRP composites sector (Adapted form [3]).

Thermoset FRP: In this type, the polymer matrix is a thermosetting resin, such as epoxy or polyester, which hardens irreversibly when cured. The main advantage of thermosetting polymers lies in their ability to maintain structural rigidity even at elevated temperatures, making them ideal for high-temperature applications. The main disadvantage is that once catalyzed, a thermosetting resin loses its ability to be reversed or re-shaped, rendering the formed thermoset composite unalterable. As a consequence, recycling thermoset composites becomes exceptionally challenging and costly. The thermoset resin itself is not recyclable due to its irreversible chemical properties. Several examples of thermosetting polymer matrices include polyester, vinyl ester, epoxy, phenolic, cyanate ester, polyurethane, polyimide, and bismaleimide. Within the FRP composites sector, the most commonly used thermoset resins are polyester, vinyl ester, and epoxy (as seen in Figure 2, according to [3]). Among them, polyester holds a dominant position, with 66% of the market share. Epoxy, on the other hand, accounts for a moderate 23% share, while vinyl ester has the smallest market presence at 5%.

Polyester resins are preferred due to their economic advantage, ease of use, and quick curing time. However, their mechanical properties require improvement when compared to vinyl esters and epoxies, which offer better performance in certain applications.

Thermoplastic FRP: In this type, the matrix material is a thermoplastic resin, capable of being melted and reformed multiple times. The most commonly used thermoplastic polymer matrices are polyamide, polyethylene, polypropylene, PEEK, thermoplastic polyimide, thermoplastic polyurethane, polycarbonate, PLA, polysulfide, and polyphenylene sulfide [7]. Thermoplastic composites offer two significant benefits in certain manufacturing applications. Firstly, compared to thermosets, many thermoplastic composites show significantly increased impact resistance. In some cases, this difference can be as remarkable as 10 times the impact resistance [8]. Secondly, another major advantage of thermoplastic composites is their inherent ability to become malleable. While raw thermoplastic resins are solid at room temperature, the application of heat and pressure during the impregnation of reinforcing fibers leads to a physical change (though not a chemical reaction resulting in a permanent, nonreversible alteration). This unique characteristic allows thermoplastic composites to be re-formed and re-shaped as needed.

The process of making thermoplastic composites malleable through heat application poses challenges due to the resin’s natural solid state. Impregnating it with reinforcing fibers requires heating the resin to its melting point and applying pressure to integrate the fibers. Subsequently, the composite needs to be cooled while still under pressure.This complex procedure necessitates the use of special tooling, techniques, and equipment, many of which are expensive. Consequently, the manufacturing process of thermoplastic composites is notably more intricate and costly compared to traditional thermoset composite manufacturing [8].

**Figure 3:**
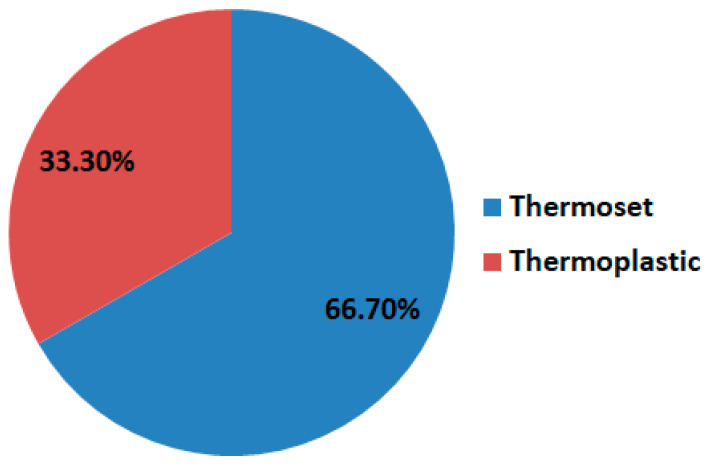
The distribution of FRP matrix (Adapted form [3]).

According to [3], compared with thermoplastics, thermoset materials represent approximately two-thirds of the FRP market share, as illustrated in Figure 3.

C. Based on the manufacturing process:

Pultruded FRP:Produced through a continuous pultrusion process where fibers and resin are pulled through a die and cured to form the composite.Pultruded FRP composites have a relatively higher fiber volume ratio, typically ranging from 60% to 75%. On the other hand, wet-laid laminates consist of several layers of fibers in multiple directions and generally have a lower fiber volume ratio [9].

Lay-up FRP: Manufactured by layering individual sheets of fiber and impregnating them with resin, followed by curing to create the composite.

Filament-wound FRP: Made by winding continuous fibers under tension around a rotating mandrel and impregnating them with resin.

D. Based on the orientation of fibers:

Unidirectional FRP: The fibers are aligned predominantly in one direction, providing high strength along that axis. They offer remarkable stiffness in the direction of the fibers, making them suitable for load-bearing applications. On the other hand, they may have lower strength and stiffness in other directions.

Bidirectional (or woven) FRP: The fibers are arranged in both longitudinal and transverse directions, offering balanced properties. Unlike unidirectional FRP, bidirectional composites exhibit similar mechanical properties in both warp and transverse directions, making them easier to analyze and design.The interlocking pattern of fibers in woven composites enhances their impact resistance compared to unidirectional materials. Regarding the disadvantages, it should be mentioned that bidirectional composites may have lower tensile strength and stiffness along specific directions compared to unidirectional composites. Also, the weaving process may result in some additional weight due to the overlapping fibers, making them slightly heavier than unidirectional materials.

Random (or chopped) FRP: Short fibers are randomly distributed in the matrix, which is suitable for improving impact resistance. Their production process is relatively simple, involving the mixing of chopped fibers with a resin matrix and molding into the desired shape. This simplicity reduces manufacturing costs, making them suitable for a range of applications where cost-effectiveness is critical. Furthermore, the random distribution of fibers in chopped FRP composites enhances their impact resistance. The entangled and interlocked fibers create a more robust structure, making these composites suitable for applications where high-impact loads are anticipated, such as automotive components, sporting goods, and protective equipment. Moreover, chopped FRP composites provide ease of application. Due to their random orientation, they can be molded into complex shapes without the need for precise fiber alignment. Despite their advantages, chopped FRP composites have certain limitations. One of the main drawbacks is their anisotropic behavior. The mechanical properties of the material vary significantly depending on the random fiber distribution, leading to unpredictable performance in different directions.Another disadvantage is that chopped FRP composites generally have lower tensile strength and stiffness compared to continuous fiber composites like unidirectional or woven FRP. This limits their use in load-bearing structures where high strength and rigidity are essential. Additionally, the random orientation of fibers may result in a rougher surface finish compared to continuous fiber composites.

E. Based on application.

FRP for construction: Used in structural components like beams and columns and in the reinforcement of buildings.

FRP for aerospace: Employed in aircraft components due to their high strength-to-weight ratio.

FRP for automotive: Utilized in vehicle parts to reduce weight and improve fuel efficiency.

FRP composites exhibit remarkable flexibility and adaptability, primarily driven by the choice of reinforcing fibers. The carbon-fiber-reinforced polymer (CFRP) stands out for its high strength, lightweight nature, corrosion resistance, and fatigue resilience, making it a preferred material for aerospace and high-performance applications. The glass-fiber-reinforced polymer (GFRP), utilizing glass fibers, offers good electrical insulation, corrosion resistance, and a cost-effective solution for various industries, including construction and automotive. The aramid-fiber-reinforced polymer (AFRP), featuring aramid fibers like Kevlar, strikes a balance between strength, stiffness, and impact resistance, finding applications in aerospace, military, and sports equipment. The basalt-fiber-reinforced polymer (BFRP) emerges as a cost-effective alternative with good corrosion resistance, biodegradability, and application versatility, particularly in construction and infrastructure. The classification based on matrix materials further enhances adaptability. Thermoset FRP, employing matrices like epoxy, polyester, and vinyl ester, maintains structural rigidity at high temperatures but faces challenges in recycling due to irreversible curing. In contrast, a thermoplastic FRP, with matrices such as polyamide and polyethylene, offers malleability and increased impact resistance, albeit with a more intricate and costly manufacturing process. The selection of manufacturing processes, fiber orientations, and application-driven choices collectively contributes to the extensive range of uses for FRP composites, spanning industries from aerospace and automotive to construction and infrastructure repair.

### 1.2. Applications of FRPs

FRPs have a wide range of applications due to their high strength-to-weight ratio, corrosion resistance, and durability. Some of the common applications of FRPs include:

1. Civil engineering and infrastructure (because they provide increased strength, load-carrying capacity, and corrosion resistance):

Strengthening and retrofitting bridges and columns with FRP wraps to increase load-carrying capacity and seismic resistance.

Repairing and reinforcing aging concrete structures, such as parking garages, using FRP composites.

Constructing lightweight pedestrian bridges and footpaths using FRP materials.

2. Aerospace and aviation (to reduce weight while maintaining structural integrity):

Manufacturing aircraft components like wings, empennages, and fuselage sections using FRP composites to reduce weight and enhance fuel efficiency.

Designing satellite components and spacecraft structures using FRP materials for their lightweight properties.

3. Automotiveindustry (to reduce vehicle weight, leading to improved fuel efficiency and performance):

Producing carbon fiber composite body panels and chassis components for high-performance sports cars and electric vehicles.

Developing lightweight FRP materials for use in automotive interiors, such as dashboards and door panels.

4. Marine and boat building (due to its resistance to corrosion from saltwater and its lightweight properties):

Fabricating boat hulls and decks using FRP materials to provide corrosion resistance and reduce overall weight.

Constructing marine wind turbine platforms using FRP composites for offshore wind farms.

5. Sports and recreation (due to their ability to enhance performance through improved strength and flexibility):

Manufacturing tennis rackets and golf club shafts using carbon-fiber-reinforced composites for increased strength and better performance.

Designing carbon fiber bicycle frames to create lightweight and rigid bicycles for competitive cycling.

6. Oil and gas industry equipment (for different equipment that require resistance to corrosion and harsh environments):

Installing FRP pipes and fittings for transporting corrosive fluids and gases in chemical processing plants and offshore installations.

Constructing FRP storage tanks to store aggressive chemicals and corrosive materials.

7. Chemical Processing (they offer excellent resistance to chemical corrosion):

Building FRP chimneys and scrubbers for chemical plants, which are resistant to acidic and corrosive fumes.

Fabricating FRP pipe systems for transporting aggressive chemicals in chemical processing facilities.

8. Electrical and electronics (due to their excellent insulating properties):

Manufacturing FRP composite insulators for electrical transmission lines to improve electrical insulation properties.

Developing FRP enclosures for electronic equipment to protect against environmental hazards and electromagnetic interference.

9. Infrastructurerepair and rehabilitation:

Strengthening damaged concrete beams and columns in bridges using FRP wraps to restore structural integrity.

Rehabilitating historical buildings with FRP composites to preserve their structural integrity and aesthetics.

10. Wind energy (due to their lightweight and high strength properties, they enable larger and more efficient blades for capturing wind energy):

Constructing wind turbine blades using advanced FRP materials to improve efficiency and increase energy capture from wind.

**Figure 4:**
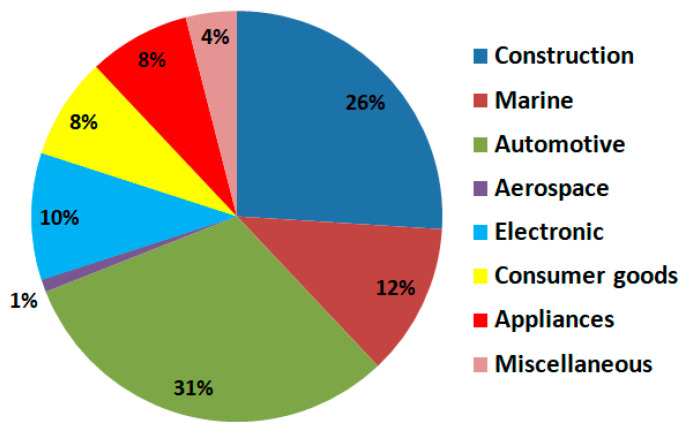
The distribution of fiber-reinforced polymer (FRP) market share across various applications (Adapted form [11]).

In Figure 4, the market share of FRP applications, according to [10,11], is presented.

Fiber-reinforced polymer (FRP) composites are materials made from a polymer matrix reinforced with engineered, man-made, or natural fibers like carbon, glass, or aramid. Some distinguishing characteristics of FRP composites mentionedin this study include the following:

- High Strength-to-Weight Ratio: FRP composites are known for their high tensile strength while being significantly lighter than metals. This makes them ideal for aerospace, automotive, and sporting goods applications where weight savings are crucial;
- Corrosion Resistance: Unlike metals, FRP composites do not corrode, making them suitable for use in corrosive environments such as chemical processing plants or marine applications;
- Flexibility in Design: FRP composites can be molded into complex shapes, which allows for design flexibility. This is significant in the aerospace and automotive industries, where aerodynamic shapes are required for performance;
- Directional Strength: The strength of FRP composites can be tailored to specific directions through the orientation of fibers. This is important in applications where the load direction is known, and the material can be designed to maximize strength in that direction;
- Good Fatigue Resistance: FRP composites have a high fatigue endurance limit, making them suitable for applications involving cyclic loads, such as bridges or rotating machinery parts;
- Thermal Properties: FRP composites have low thermal conductivity and good thermal insulation properties. This characteristic is crucial in applications where thermal insulation is required or in situations where the material must withstand extreme temperature variations without deforming;
- Electrical Properties: FRPs are generally non-conductive, which makes them useful in electrical insulation applications and for use in electromagnetic-sensitive environments.

The significance of these characteristics in material selection and application is that they allow engineers and designers to tailor materials to the specific needs of their application. For example, in a corrosive environment, the corrosion resistance of FRP might be the deciding factor, while in an aerospace application, the high strength-to-weight ratio would be most critical. Understanding the specific environment and demands of the application is essential to making the best material choice.

## 2. Historical Background of FRPs

The utilization of composite materials, owing to their intriguing physical and mechanical attributes, has become prevalent across numerous engineering domains. These materials are now integral in advanced applications within civil, mechanical, aerospace, and biomedical fields. Interestingly, the concept of combining two or more elemental materials to create composite solids has been employed since the inception of conscious or subconscious structural design processes [12,13,14].

Historically, ancient Jewish workers, while under Pharaoh rule, blended chopped straws with clay to craft bricks with enhanced mechanical strength. Japanese samurai warriors employed laminated metals, utilizing steel strips for strength and iron strips for flexibility when forging their swords. Around 1200 AD, the Mongols invented the first composite bow by combining wood, bone, and animal glue [15].

In the realm of modern advanced composite materials, particularly during the plastic era, one of the most crucial types, from a structural perspective, is fiber-reinforced composites. In this category, the use of fibers with distinct physical characteristics strategically arranged within a matrix material facilitates the creation of functional materials with desired levels of strength and stiffness. These materials can also exhibit advantageous chemical and physical properties. Typically, the matrix consists of polymeric resins like epoxy, vinylester, or polyester thermosetting plastics. However, metals, such as aluminum, or mortar/concrete, are also employed for specific structural applications [13].

Regarding commonly used fiber-reinforced materials, the primary fiber materials are glass, carbon, and aramid compounds, although other fibers find application in structural contexts [16]. These include vegetable fibers (e.g., cotton, hemp, jute, flax), wood fibers (distinct from vegetable fibers), and mineral fibers (e.g., asbestos). The history of textile fibers spans millennia, with the utilization of wool dating back over 4000 years. In stark contrast, the inception of the synthetic fiber industry can be traced to the initial commercial production of rayon in 1910. The 1950s and 1960s witnessed a significant surge in technological advancements within the synthetic fiber sector.

It is worth noting that fiber-reinforced polymers (FRP) are not a recent innovation. In fact, over a century ago, Leo Baekeland, an American chemist of Belgian origin, pioneered an FRP with his creation known as Bakelite. This material was groundbreaking due to its distinctive properties [6].

**Figure 5:**
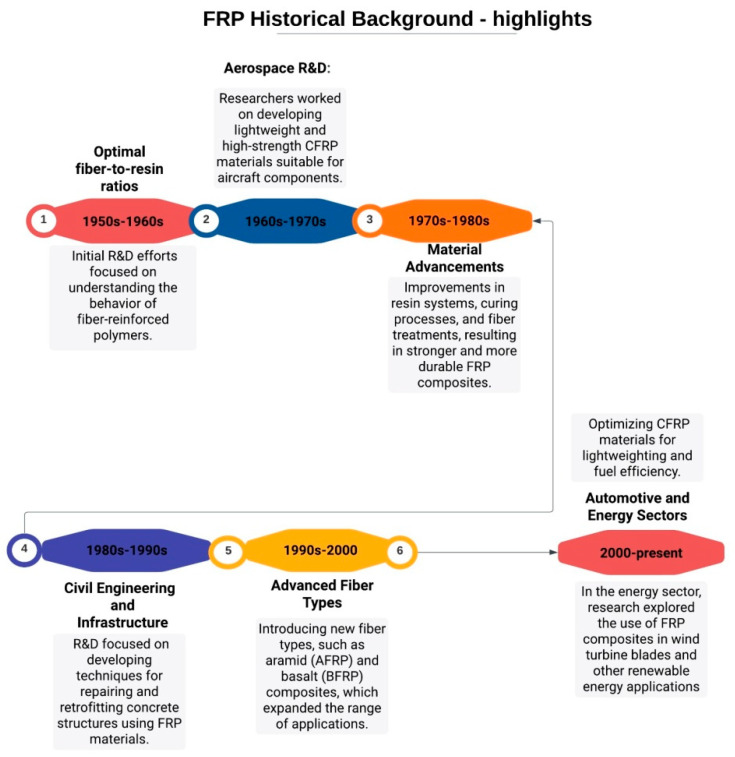
Milestones of the FRP materials evolution across time.

In the year 1935, Owens Corning unveiled the inaugural glass fiber, thereby heralding the inception of the fiber-reinforced polymer (FRP) industry. A year thereafter, in 1936, patents were granted for unsaturated polyester resins. Thanks to their curing capabilities, unsaturated polyester resins remain the predominant choice for manufacturing resins today [4]. Figure 5 presents a historical timeline that encapsulates vital developments regarding fiber-reinforced polymer (FRP) composites. This timeline offers some highlights and an overview of the evolution of FRP compositestudies, from their early experimental stages to their widespread adoption in diverse industries.

Figure 5 shows the continuous innovation and research efforts that have propelled FRP composites into prominent roles in fields ranging from aerospace to civil engineering.

1940s–1950s:

Early Research: The concept of reinforcing polymers with fibers began to emerge in the mid-20th century. Early experiments involved using materials like fiberglass to reinforce plastic resins.

1950s–1960s:

Development of Fiberglass: The development of fiberglass, which is a glass-fiber-reinforced polymer (GFRP), gained momentum during this period. Companies like Owens Corning and others started producing fiberglass for various applications, including boats and automotive parts.

1960s–1970s:

Aerospace Applications: CFRP (carbon-fiber-reinforced polymer) composites were introduced in the aerospace industry, primarily in the form of carbon-fiber-reinforced epoxy composites. These materials offered significant weight savings for aircraft components.

1970s–1980s:

Growth in the Marine Industry: The GFRP gained popularity in the marine industry due to its corrosion resistance and lightweight properties. It became widely used for boat hulls and structures.

1980s–1990s:

Civil Engineering and Infrastructure: The use of FRP composites in civil engineering and infrastructure projects began to grow. Applications included bridge deck reinforcement and the repair of concrete structures.

1990s–Present:

Advancements in Materials: Ongoing research led to the development of advanced materials like aramid- (AFRP) and basalt- (BFRP) fiber-reinforced polymers, expanding the range of applications for FRP composites.

2000s–Present:

Automotive Industry: CFRP composites found increasing use in high-end and performance vehicles due to their lightweight properties and potential for improved fuel efficiency and performance.

2010s–Present:

Construction and Architecture: FRP materials gained popularity in the construction and architectural sectors. They were used for applications such as building facades, cladding, and reinforcement of concrete structures.

Ongoing Innovations: Research and development in FRP composites continue, with a focus on improving materials, manufacturing processes, and recycling methods. Efforts are also being made to reduce costs and environmental impacts.

Future Prospects: FRP composites are expected to play a significant role in sustainable engineering and construction practices, as they offer the potential for reduced energy consumption and greenhouse gas emissions.

In summary, the history of FRP composites spans several decades and has seen a gradual expansion from early experimentation to widespread use in industries such as aerospace, automotive, marine, construction, and civil engineering. The ongoing development of advanced materials and manufacturing techniques suggests that FRP composites will continue to have a growing impact in various sectors in the future.

## 3. Manufacturing Technologies of FRPs

The fabrication of FRP composite structures is a complex process, including a variety of methods, depending on the type of reinforcement and the intended application of the product [11,17,18,19]. The production process comprises two essential steps: first, the creation of fiber preforms, followed by the reinforcement of these preforms with the matrix material. 

**Figure 6:**
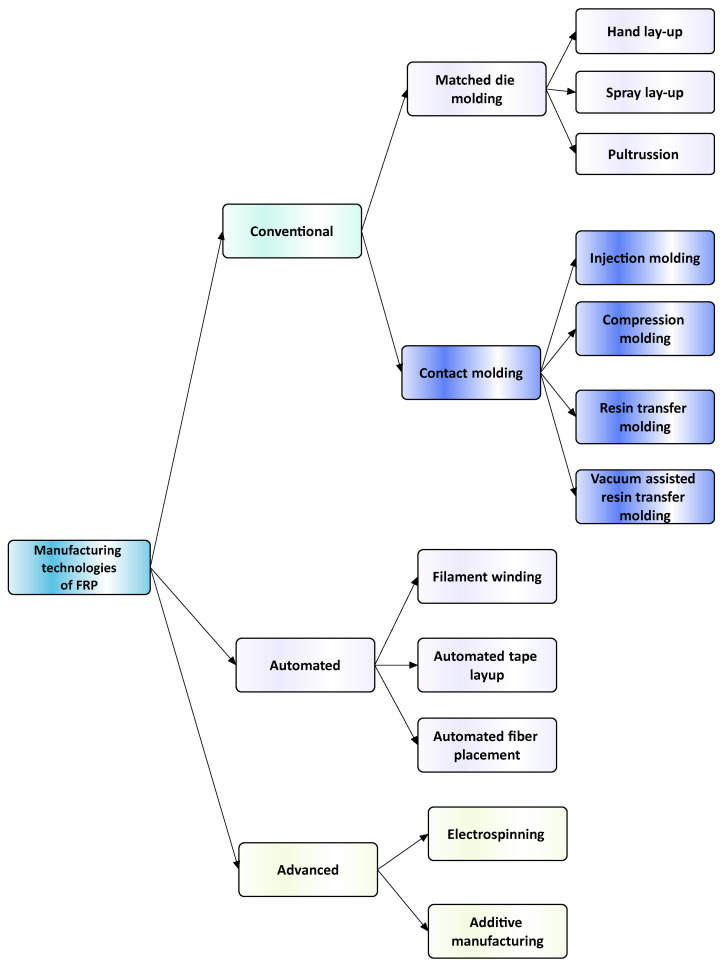
Classification of FRP manufacturing technologies (Adapted form [20]).

Figure 6 illustrates the classification of the main composite manufacturing process techniques into three groups: conventional, automated, and advanced manufacturing techniques [20,21,22].

Figure 6 illustrates the classification of the main composite manufacturing process techniques into three groups, conventional, automated, and advanced manufacturing techniques, and Table 3 presents a summarized description of each manufacturing technology, including the process schematization, the application field, and the advantages and disadvantages.In this manuscript, we added the key features for each category. Conventional manufacturing methods for FRP are characterized by their inherent flexibility and versatility. Processes like lay-up and filament winding allow for customized designs and intricate lay-up patterns, making them adaptable to diverse project requirements. However, these methods heavily rely on manual labor, which can introduce variability in the final product and may not be as efficient for large-scale production. In contrast, automated manufacturing technologies, exemplified by processes like filament winding, automated fiber placement, and automated tape lay-up, emphasize efficiency and repeatability. Automation reduces dependence on manual labor, leading to consistent and reliable products. These methods improve production rates while maintaining a degree of flexibility, making them suitable for applications where precision and efficiency are essential. Moving further on the technological spectrum, advanced manufacturing technologies like electrospinning and additive manufacturing share a common emphasis on precision and customization in the fabrication of FRP. Electrospinning stands out for its ability to produce ultrafine fibers with remarkable precision, offering control over diameter, alignment, and composition. This characteristic aligns with additive manufacturing, which is defined by its layered construction approach, enabling the additive buildup of intricate designs and complex geometries with precision. Both methods contribute to the development of adapted FRP composites, allowing for the customization of mechanical and functional properties. While electrospinning excels in creating materials with a high surface-to-volume ratio, additive manufacturing’s key feature lies in its versatility across various materials, rapid prototyping capabilities, and the ability to create complex, customized FRP structures, making them valuable advancements in the field of composite material fabrication.

Table 3 presents a summarized description of each manufacturing technology, including the FRP type used, the application field, and the advantages and disadvantages. 

The manufacturing process is a crucial factor in the classification of FRP composites, significantly influencing their final properties and applications. Various processes, such as pultrusion, lay-up, and filament winding, contribute distinct characteristics to the resulting composites. For example, pultruded FRP composites, produced through a continuous process, tend to have a higher fiber volume ratio, offering enhanced strength and stiffness. On the other hand, lay-up processes involve layering individual sheets of fiber and resin, providing flexibility in design and allowing for specific lay-up requirements. Filament winding, where continuous fibers are wound around a mandrel, results in composites with excellent strength and is commonly used for cylindrical structures. The choice of manufacturing process, therefore, allows adapting FRP composites to meet diverse application needs, balancing factors such as strength, flexibility, and cost-effectiveness.

## 4. Insights into Material Characteristics and Design Guidelines

FRP composites are advanced materials made up of fibers, resins, fillers, and additives. These components work together to give FRP special properties. The fibers provide strength, the resins protect them from damage, and fillers help control costs and shrinkage, so this combination creates FRPs’ unique characteristics [27].

**Figure 7:**
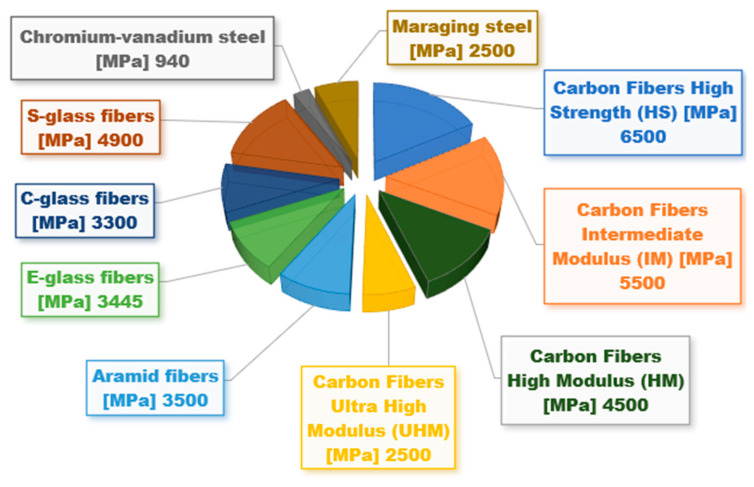
Comparison regarding the tensile strength of different materials used in industry.

Four primary materials, namely carbon, glass, aramid, and basalt, are commonly employed in engineering to create fibers. These fibers are used to form CFRPs, GFRPs, AFRPs, and BFRPs. Figure 7 illustrates a comparison regarding the tensile strength of different materials used in industry

Reference [28] provides an in-depth examination of four widely used fiber-reinforced polymer composites (AFRPs, BFRPs, CFRPs, GFRPs) by evaluating crucial material properties, as can be seen in Table 4 [28].

Table 5 presents the key mechanical properties of high-performance sheet materials, including aramid, E-glass, carbon, PBO, Dyneema, PET, PEN, and PAF, providing a comprehensive overview of their mechanical characteristics [28].

Table 6 provides a comprehensive overview of the typical properties of CFRP (carbon-fiber-reinforced polymer) composites, including variations such as pitch carbon, polyacrylonitrile carbon, common CFRP, high-modulus CFRP, and high-strength CFRP. The properties examined encompass a range of key characteristics crucial for material selection and application.

Table 7 showcases the distinctive attributes of various glass fiber reinforcements, including E-glass, S-glass, C-glass, and AR-glass, which have been revealed through a thorough examination of their properties. This comprehensive analysis offers valuable insights into the specialized merits and applications of each type.

Table 8 outlines the unique properties of diverse aramid fibers, including Twaron, Twaron HM, Technora H, Kevlar 29, Kevlar 49, and Kevlar 149, providing valuable insights into their specific advantages and applications within high-performance materials.

Table 9 illustrates the characteristic properties of thermoset resins used as matrices for FRPs, including epoxy, vinylester, and polyesters, as outlined in reference [29]. This comparative analysis offers valuable insights into their suitability and performance in composite materials.

The mechanical attributes of FRP composites are influenced by several factors, including the balance between fibers and matrix materials, the manufacturing process, individual material properties, and the alignment of fibers within the matrix. Fibers can be configured in various ways: ina“Continuous Form” (where fibers are straight, long, and parallel to each other), in a “Woven Form” (forming a cloth-like structure for strength in multiple directions), or in a “Chopped Form” (where fibers are irregularly arranged, discontinuous, and shorter, often referred to as fiberglass) [31].

In relation to tensile strength, as elucidated by the subsequent bibliographic references [32,33,34,35,36,37,38,39,40,41], the ensuing facets have been discerned:

The CFRP offers lower weight and higher tensile stress compared to other FRPs, leading to significant weight reduction and increased span lengths for prestressed components.

The BFRP has exceptional tensile strength and elongation at breaking.The GFRP enhances tensile strength by 36% in hybrid FRPs compared to the BFRP, surpassing PBO by 2.56%.

Tensile strength for the vinyl ester matrix is minimally affected, while for the polyester matrix, it decreases by a significant 80% in GFRPs.

Tensile strengths for GFRPs with epoxy-based and polyester-based matrices decrease by approximately 22.8% and 19.71%, respectively, when filled with rice husk and wheat husk.

Overall, hybrid GFRPs display more variable tensile strength compared to CFRPs or a GFRP alone.

The primary determinants of compressive strength, as outlined by [42,43,44,45,46,47,48], encompass the following:

CFRPs and AFRPs have the highest and lowest compressive strengths among typical FRP composites.

When basalt fibers are used on the surface or in layered formations (creating a sandwich-like structure), they significantly increase strength.

Composites with epoxy-based fibers tend to have higher compressive strength compared to those with polyester-based fibers. This means that composites with epoxy-based materials are generally stronger.

Wrapping hollow columns with CFRP layers (one layer or three layers) improves strength by 66% and 123%, respectively. In comparison, wrapping with a GFRP only improves strength by 36% and 105% for the same layering.

When hollow columns are filled with concrete and wrapped with a CFRP (three layers), the strength increases by 154%. For GFRP wrapping with the same layering, the strength increases by 144%. This shows that filling columns with concrete and adding FRP wrapping greatly enhances their strength.

Using FRPs to strengthen ultra-high-performance concrete (UHPC) increases its compressive strength by at least 115% compared to regular UHPC.

Sandwich structures reinforced with CFRPs have approximately 24.68% higher compressive strength compared to non-reinforced structures.

Increasing the thickness of FRP layers effectively boosts the compressive strength of reinforced concrete elements in specific areas.

Regarding shear strength, pivotal factors, as outlined by [49,50,51,52,53,54], involve the following:

Absorbing moisture in epoxy weakens shear strength in RC elements. To counter shear stresses, FRP-reinforced structures require stirrups or ties or rely solely on concrete strength (as in RC tanks).

FRP rebars generally have lower stiffness, necessitating deeper elements or extra reinforcement to reduce deflection and crack widths.

ACI 318 guides the design of shear strength for FRPs, while ACI 440 does not allow dowel action for FRP rebars, in contrast to steel rebars.

Textiles are engineered to optimize shear beam strength in 45-degree directions, yet rosette strain gages reveal these materials surpass shear cracks in strength.

Table 10 delineates the essential characteristics of GRP pipes, with particular emphasis on their hoop and axial properties, as documented in a 2013 research report [55], offering valuable insights into their performance and applicability within composite materials.

**Figure 8:**
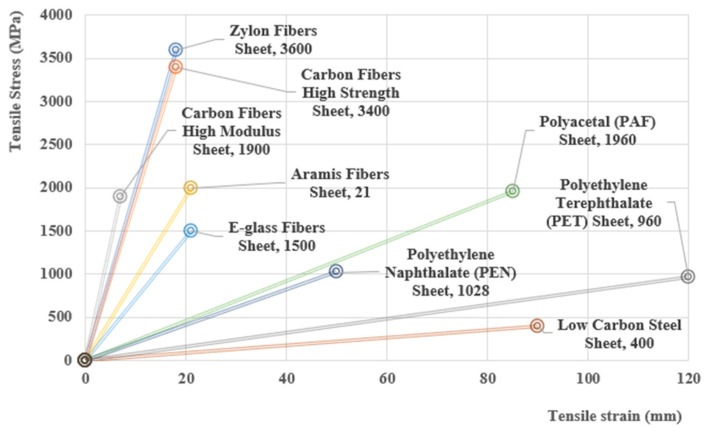
Stress and strain relationships of different fiber composite.

Figure 8 depicts the stress–strain relationships observed in various fiber composites, providing a visual representation of their mechanical behavior and performance under applied loads [56].

ISO 14692 [57] draws upon ASME B 31.3 guidelines to define system load cases like sustained, expansion, and occasional loads. Additionally, it relies on BS7159 for system design parameters, encompassing flexibility factors, stress intensity factors (SIF), and pressure stress multipliers. These standards collectively inform the approach taken in evaluating the integrity of GRP process piping systems and pipelines, considering factors such as pipework flexibility, layout complexity, pipe supports, pipe diameter, temperature fluctuations, and system criticality and failure risk assessment. Piping stress engineers must conduct a comprehensive analysis to ascertain if flexibility analysis is warranted.

This entails determining various loading conditions—internal or external pressure, thermal, occasional, and support loads—and subsequently assessing the associated stresses and loads, which are then compared with established allowable limits.

Laminated FRP structures exhibit distinct behavior influenced not only by the mechanical attributes of their constituent elements, such as strength, elastic modulus, and stress limits but also by the specific geometry imposed on each ply within the laminate.

**Figure 9:**
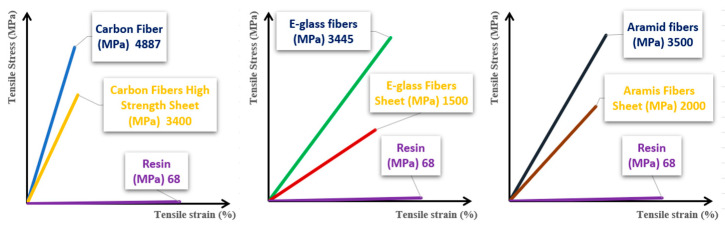
Comparative influence of fiber and resin on FRP composite performance.

In general, the reinforcement fibers tend to possess superior elastic properties in comparison to the polymeric matrix. Consequently, the overall properties of the composite structure predominantly hinge on the characteristics of the reinforcement (see Figure 9).

In order to determine the characteristics of GRP/FRP materials, it can be used the standards [58,59,60,61,62,63,64,65,66,67,68,69,70,71,72,73,74,75,76,77,78,79,80,81,82,83,84,85,86,87,88,89,90,91].

The tensile strength of FRP composites is often much higher than that of traditional materials like steel or aluminum when compared on a weight basis. This is largely due to the high tensile strength of the reinforcing fibers, such as carbon or glass fibers.While not as high as tensile strength, the compressive strength of FRP composites is adequate for many structural applications, particularly when designed with appropriate fiber orientations.FRP can be designed to absorb impact energy through damage mechanisms such as fiber breakage and delamination, which can prevent catastrophic failure.The stiffness of an FRP composite, measured by its modulus of elasticity, is determined by the type of fibers used and their volume fraction. High-stiffness fibers like carbon can provide the rigidity necessary for structural applications.In terms of corrosion resistance, an FRP’s polymer matrix is inert to many corrosive substances, which gives FRP composites an advantage in harsh chemical or saline environments, leading to a longer lifespan with less maintenance. On the other hand, UV inhibitors and protective coatings can be added to FRP composites to enhance their resistance to sunlight and weathering, making them suitable for outdoor applications. Furthermore, the lower density of FRP composites compared to metals leads to significant weight savings, which is especially beneficial in the automotive and aerospace industries for fuel efficiency and performance. FRPs have low thermal conductivity, which makes them good insulators. This property is vital in applications where thermal bridging must be minimized, such as in building envelopes, while FRPs typically have a lower coefficient of thermal expansion than metals, which means they have less dimensional change with temperature fluctuations. Also, the dielectric properties of FRP composites make them suitable for electrical insulation applications, as well as for use in structures where electrical conductivity could be hazardous.FRP composites can be molded into complex shapes and sizes, which allows for integrated designs and the reduction of joint and fastener needs.

These material characteristics make FRP composites highly versatile and enable their use in a diverse range of applications, from simple consumer products to advanced aerospace components. The ability to customize the fiber and matrix combinations allows for optimization of performance to meet specific requirements, whether it is maximizing strength for structural components, ensuring durability for outdoor use, or achieving a balance between strength and weight for mobility and transport applications.

## 5. Comprehensive Design Guidelines for Fiber-Reinforced Polymer (FRP) Structures

Designing structures with fiberglass-reinforced plastic (FRP) materials requires adherence to specific standards and guidelines to ensure safety, reliability, and compliance with industry best practices. The following are some of the key standards and codes that are commonly used for FRP design:

ACI 440.1R-15 (American Concrete Institute) [92].

This document provides comprehensive guidance on using FRP materials as reinforcement in concrete structures. It covers design considerations, material properties, and construction practices.

It addresses various types of FRP reinforcement, such as bars, grids, and sheets.

The standard offers design examples and calculations to assist engineers in designing FRP-reinforced concrete members.

ACI 440.6-08 (American Concrete Institute) [54].

This standard specifies the requirements for carbon and glass fiber-reinforced polymer bars used as reinforcement in concrete structures.

It outlines the materials, fabrication, and testing requirements for FRP bars, ensuring that they meet the necessary strength and durability criteria.

ACI 440.4R-04 (American Concrete Institute) [93].

This guide focuses on the prestressing of concrete structures using FRP tendons.

It covers design considerations, material properties, and construction practices related to prestressed concrete elements reinforced with FRPs.

ASTM D7205 (ASTM International) [94]:

ASTM D7205 provides standardized testing methods for determining the mechanical properties of FRP composites used in civil engineering applications.

It covers tensile, compressive, flexural, and shear testing of FRP materials, ensuring accurate characterization of their performance.

ASCE Manuals and Reports on Engineering Practice No. 124.

This manual is a valuable resource for engineers and designers working with FRP-reinforced concrete structures.

It offers detailed information on the design, analysis, and construction of such structures, including case studies and practical examples.

ISO 10406-1 and ISO 10406-2 (International Organization for Standardization) [95,96].

ISO 10406 standards provide guidelines for the design, manufacturing, and testing of pultruded FRP profiles for structural applications.

Part 1 covers material properties, while Part 2 addresses design considerations and performance requirements.

ACMA FRP Composite Bridge Committee.

This committee within the American Composites Manufacturers Association focuses on developing standards and guidelines specifically tailored to FRP bridges.

Their standards cover topics such as design methodologies, manufacturing processes, quality control, and installation practices for FRP bridge components.

AASHTO LRFD Bridge Design Specifications (American Association of State Highway and Transportation Officials) [97].

These specifications are widely used in the United States for designing bridges, including those constructed with FRP materials.

Engineers should refer to relevant sections within AASHTO LRFD that pertain to FRP design, detailing, and testing requirements for bridge components.

Standards and codes that are commonly used for FRP piping (in petroleum and natural gas industries).

ISO 14692-1:Petroleum and natural gas industries—Glass-reinforced plastics (GRP) piping Part 1: Vocabulary, symbols, applications and materials [98];

ISO 14692-2: Petroleum and natural gas industries—Glass-reinforced plastics (GRP) piping Part 2: Qualification and manufacture [99];

ISO 14692-3: Petroleum and natural gas industries—Glass-reinforced plastics (GRP) piping Part 3: System design [100];

ISO 14692-4: Petroleum and natural gas industries—Glass-reinforced plastics (GRP) piping Part 4: Fabrication, installation and operation [57].

ASME B31.3: The ASME B31.3 code provides guidelines for the design, construction, and inspection of process piping systems used in the chemical, petroleum, and allied industries, including those made of FRP [101].

API 15LR: Published by the American Petroleum Institute (API), this standard addresses the design, fabrication, installation, inspection, and testing of fiberglass-reinforced thermosetting plastic (FRP) piping systems for offshore platforms [102].

ASTM D2996: ASTM D2996 provides standard specifications for filament-wound “fiberglass” (glass-fiber-reinforced thermosetting resin) pipes, which are commonly used in FRP piping systems [88].

ASTM D3262: This ASTM standard covers “fiberglass” (glass-fiber-reinforced thermosetting resin) sewer and industrial pressure pipes, which may also be used in the petroleum and natural gas industries [103].

NACE MR0175/ISO 15156: NACE International’s MR0175/ISO 15156 standard addresses the selection of materials for equipment used in oil and gas production environments, including FRP piping, where sour (containing hydrogen sulfide) conditions may be encountered [104].

AWWA M45: The American Water Works Association (AWWA) publishes standards for various types of pipes. AWWA M45 provides guidelines for the design and installation of fiberglass pipes and fittings, including those used in water and wastewater services for industries like petroleum refining.

ASTM D4024: ASTM D4024 provides guidelines for designing and specifying glass-fiber-reinforced thermosetting resin pipe, which is commonly used in FRP piping systems.

API 15HR: API 15HR is an API standard specifically addressing the design, materials, fabrication, testing, and inspection of FRP piping systems for use in oil and gas production, processing, and transportation.

ASME RTP-1: While primarily focused on equipment such as tanks and vessels, ASME RTP-1 also includes guidelines for the design, fabrication, and inspection of FRP piping systems in various industries, including petroleum and gas.

BS 7159:1989 British Standard Code of Practice for Design and Construction of Glass-reinforced Plastics (GRP) Piping Systems for Individual Plants or Sites.

### Methods for Assessing the Flexibility of FRP/GRP/GRE Pipes

The assessment of the performance of fiber-reinforced plastic pipes under combined pressure and temperature loads is of paramount importance to guarantee their structural integrity and operational efficiency. Extensive research has indicated that FRP exhibits exceptional resistance to corrosion and possesses a remarkable ability to withstand mechanical stresses.

However, when subjected to simultaneous pressure and temperature variations, the behavior of FRP pipes can be influenced significantly.

It is crucial to recognize that pressure and temperature fluctuations can have a substantial impact on several key factors associated with FRP pipes. These factors include but are not limited to the following.

Thermal Expansion:The composite nature of FRP pipes means that they may experience significant thermal expansion and contraction in response to temperature changes. This expansion can affect the overall dimensions and shape of the pipe, potentially leading to structural issues if not properly managed.

Material Stiffness: The mechanical properties of FRP, such as its modulus of elasticity, can be temperature-dependent. Elevated temperatures may reduce the stiffness of the material, affecting its ability to withstand external loads and maintain its shape.

Stability: The combination of pressure and temperature can impact the overall stability of FRP pipes, potentially leading to buckling or deformation under certain conditions.

To ensure the safe and efficient utilization of FRP pipes in specific applications, comprehensive evaluations must be conducted. These evaluations should encompass finite element analysis, stress and strain assessments, and computational simulations to predict the behavior of FRP pipes under various operating conditions. Furthermore, the outcomes of these assessments will play a pivotal role in guiding the design and engineering of FRP piping systems, allowing for the optimization of material selection, reinforcement strategies, and thermal insulation. Ultimately, these efforts will ensure the reliable functionality and safety of FRP pipes, particularly in industries where they are commonly employed, such as chemical processing, oil and gas, and water treatment.

ISO 14692 is an international standard widely used in the petrochemical and natural gas industries for fiber-reinforced polymer (FRP) pipelines. This standard provides detailed guidance on the design, manufacturing, installation, and maintenance of FRP pipelines, including stress analyses.In the context of stress analysis, ISO 14692 offers a methodology for assessing the behavior of FRP pipelines under various loads and operating conditions. The standard defines relevant criteria and parameters for determining the stresses and deformations to which an FRP pipeline may be subjected, as well as for evaluating material compatibility. ISO 14692 contains recommendations and specific requirements for stress analysis in FRP pipelines, covering the following aspects:

Internal Pressure: The standard provides guidelines for calculating and evaluating stresses generated by internal pressure acting on the pipelines. It includes methods for determining the required wall thickness of the pipeline and assessing safety based on the maximum working pressure.

Operating Temperature: ISO 14692 contains recommendations for assessing thermal stresses in FRP pipelines based on operating temperatures. It covers aspects such as thermal cohesion, dimensional changes, and material expansion.

External Loads: The standard also addresses the analysis of stresses caused by external loads, such as mechanical loads, vibrations, earthquakes, or wind loads. It provides guidance on determining external loads and evaluating the stresses generated by them in FRP pipelines.

ISO 14692 also includes requirements and recommendations for verifying and validating the results of stress analyses, as well as tests and inspections necessary to ensure the quality and safety of FRP pipelines.By using ISO 14692 in stress analyses of FRP pipelines, it ensures that these pipelines are designed and manufactured to withstand various loads and operating conditions, thus ensuring their safety and reliability in industrial applications.

Materials like FRP (fiber-reinforced polymer) fall under the category of orthotropic materials, and they fundamentally differ from materials made of steel, which are isotropic. As can be observed, for orthotropic materials (such as FRP and GRP), the modulus of elasticity in the axial direction differs from that in the circumferential direction. Similarly, the Poisson’s ratio in the axial direction differs from that in the circumferential direction.

Orthotropic materials exhibit distinct mechanical properties along different axes, which make them anisotropic. This anisotropy arises from the orientation and alignment of the reinforcing fibers within the polymer matrix. In the case of FRP and GRP, these reinforcing fibers, often composed of materials like glass or carbon, provide the materials with their unique properties.

For example, in FRP composites, the modulus of elasticity is typically higher in the direction aligned with the fibers (axial direction) compared to the direction perpendicular to the fibers (circumferential direction). This is due to the enhanced stiffness provided by the aligned fibers. Consequently, FRP materials are excellent candidates for applications where directional stiffness and strength are critical, such as in the construction of composite structures like pipes, pressure vessels, or aerospace components.

Additionally, the Poisson’s ratio, which describes the ratio of lateral strain to axial strain when a material is subjected to uniaxial loading, can vary in different directions for orthotropic materials. In FRP materials, the Poisson’s ratio in the axial direction may differ from that in the circumferential direction, reflecting the different deformation behavior along these axes.

Understanding these orthotropic properties is crucial in the design and analysis of structures and components made from FRP materials. Engineers must consider these directional variations in mechanical properties when predicting the behavior of such materials under different loading conditions, ensuring that the designs are optimized for performance and safety.

The shear modulus (1), also known as the modulus of rigidity or shear modulus of elasticity, is a measure of a material’s resistance to deformation caused by shear forces. It is typically denoted by the symbol “*G*” and is calculated as the ratio of the applied shear force to the resulting shear angle, divided by the product of the material’s volume and its displacement under shear.The shear modulus is an important characteristic of materials and is used in engineering to assess a material’s behavior in applications involving shear forces, such as in shafts and motion transmission elements.

(1) $$G=\frac{M_{t}\cdot L}{∆\varphi \cdot 2l}$$

where

*G* is the shear modulus of the material;

*M_t_* is the applied torsional moment;

*L* is the length of the element subjected to torsion;

∆φ represents the torsional angle or torsional deformation;

*l* is the length of the material undergoing deformation due to torsion.

The shear modulus (G), in the case of FRP pipes, is calculated using the following equation:

(2) $$G=\frac{E_{a}\cdot E_{h}}{E_{a}+E_{h}+2{\cdot E}_{a}\cdot \nu_{ha}}$$

where

E_a_, is the Young’s modulus in the axial direction;

E_h,_ is the Young’s modulus in the hoop direction;

ν_ha,_ is the Poisson’s ratio in the axial–hoop direction.

Formula (2) is used to determine the stiffness or elastic behavior of a material when subjected to forces or deformations in both the axial and transverse directions. It takes into account the material’s Young’s modulus values in both directions as well as the Poisson’s ratio for axial–transverse deformation.

The relationship between Poisson’s ratio and the elastic moduli (axial and hoop) in fiber-reinforced polymer (FRP) materials is expressed by the following equation:

E_h_/E_a_ = ν_ha_/ν_ah_ (3)

This equation describes a fundamental aspect of the mechanical behavior of FRP materials, where E_h_ represents the elastic modulus in the hoop direction, which measures the material’s resistance to deformation when subjected to forces applied tangentially to its surface, E_a_ represents the elastic modulus in the axial direction, which measures the material’s resistance to deformation when subjected to forces applied along its length, ν_ha_ represents the Poisson’s ratio in the axial–hoop direction. Poisson’s ratio is a dimensionless material property that describes how a material responds to deformation. In this case, it specifically addresses how the material’s width changes when it is subjected to axial stretching, ν_ah_ represents the Poisson’s ratio in the hoop–axial direction. This is essentially the opposite of ν_ha_ and describes how the material’s length changes when it is subjected to transverse compression.

The equation tells us that the ratio of the hoop elastic modulus to the axial elastic modulus is equal to the ratio of the axial–hoop Poisson’s ratio to the hoop–axial Poisson’s ratio. This relationship is significant because it helps characterize how FRP materials behave when subjected to different types of loading.

The axial (and hoop) specific strain is determined, in the case of FRP pipes, using the following equations:

(4) $$\varepsilon_{a}=\frac{\sigma_{a}}{E_{a}}-\nu_{ha}\cdot \frac{\sigma_{h}}{E_{h}}$$

(5) $$\varepsilon_{h}=\frac{\sigma_{h}}{E_{h}}-\nu_{ha}\cdot \frac{\sigma_{a}}{E_{a}}$$

In practical terms, understanding this relationship is crucial for designing and analyzing structures and components made from FRP materials. Engineers and researchers can use this equation to predict how FRP materials will respond to various mechanical loads, ensuring the safety and efficiency of FRP-based applications in various industries.In many engineering and structural analysis applications involving pipe-like elements, the circumferential specific strain is not explicitly considered for simplicity because it does not significantly affect the overall behavior of the structure in most cases. In the next list, we provide a more detailed explanation of why this is the case:

Assumption of Thin-Walled Structures: In many situations, pipes and cylindrical structures are thin-walled. This means that the wall thickness is much smaller compared to the radius of the structure. When the wall thickness is significantly smaller than the radius, circumferential strains are typically very small, and thus, they are often neglected.

Simplified Analysis: Ignoring circumferential strains simplifies the analysis of these structures, making it easier to calculate and predict their behavior under various loading conditions. Engineers can focus on the axial and radial components of stress and strain, which are typically the most significant factors in such structures.

Tensile and Compressive Stresses: In many cases, pipes are subjected to axial loads (tensile or compressive) or internal pressure. The axial and radial stresses resulting from these loads are the primary factors that engineers need to consider when designing or analyzing the structural integrity of the pipe. Circumferential strains may only become significant when there are additional factors, such as local geometric irregularities or discontinuities.

Simplified Design Codes: Many design codes and standards for piping and cylindrical structures are based on simplified assumptions that exclude circumferential strains. These codes have been developed over decades of engineering practice and have proven to be adequate for ensuring the safety and reliability of such structures.

Exceptions: While circumferential strains are often neglected, there are cases where they must be considered. These exceptions typically involve specific situations where circumferential stresses become significant, such as in the design of thick-walled pressure vessels or when dealing with local stress concentrations.

The mechanical characteristics of FRP/GRE (fiber-reinforced polymer/glass-reinforced epoxy) pipes can vary from one manufacturer to another. For example, in Table 11 and Table 12, mechanical characteristics are presented according to Wavistrong Engineering.

Pipe systems made of fiber-reinforced polymer (FRP) exhibit variations in deformation characteristics depending on whether they are classified as restrained or unrestrained. This differentiation has significant implications for the behavior of these pipe systems in various applications. The difference between restrained and unrestrained pipes lies in how they are supported and whether they are allowed to move longitudinally within a system. Restraint pipes are anchored or constrained at specific points to prevent movement, while unrestrained pipes are allowed to move freely within the constraints of their supports. The choice between restrained and unrestrained pipes depends on the specific requirements of the application, including factors such as system design, temperature variations, fluid pressure, and potential external forces like seismic activity.

The specific axial deformation in the case of unrestrained pipe systems is calculated according to the following equation:

(6) $$\varepsilon_{a}=\frac{\sigma_{a}}{E_{a}}-\nu_{ha}\frac{\sigma_{h}}{E_{h}}=\frac{\alpha ∆TE_{a}+0.5\sigma_{h}}{E_{a}}-\nu_{ha}\frac{\sigma_{h}}{E_{h}}=\alpha ∆T+(0.5-\nu_{ha})\frac{\sigma_{h}}{E_{h}}$$

The elongation in the axial direction (FPR/GRP/GRE unrestrained) is calculated using the following equation:

(7) $$∆L=\alpha ∆TL+\left(0.5-\nu_{ha}\right)\frac{\sigma_{h}}{E_{h}}L$$

In the case of restrained FRP pipes, the axial stress is calculated according to the following equation:

(8) $$\sigma_{a}=\varepsilon_{a}E_{a}+\nu_{ha}\sigma_{h}\frac{E_{a}}{E_{h}}=-\alpha ∆TE_{a}+\nu_{ha}\sigma_{h}\frac{E_{a}}{E_{h}}$$

**Figure 10:**
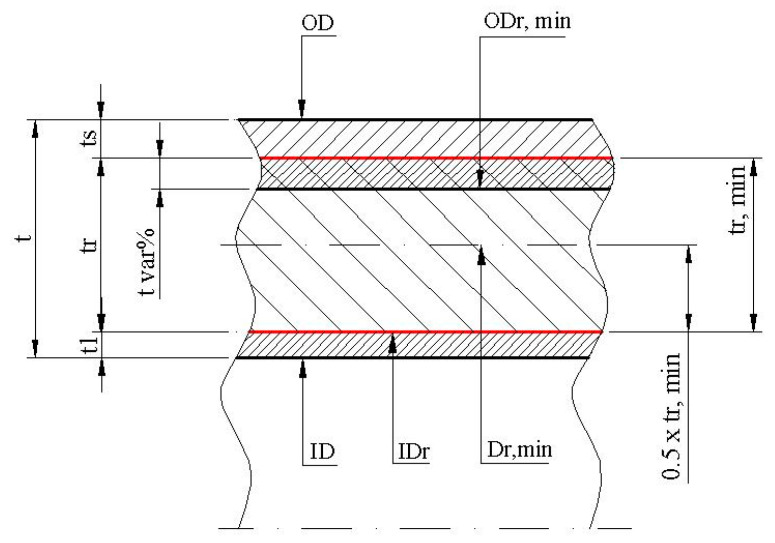
The dimensions that make up the wall thickness (Adapted form [98]).

Taking into account reference [16], the dimensions that are involved in the assessment of wall thickness in the case of FRP/GRP/GRE-type pipes are presented in Figure 10 [98].

(9) $$t_{r}=t-t_{1}-t_{2}$$

(10) $$t=\frac{OD-ID}{2}$$

(11) $${ID}_{r}=ID+2t_{1}$$

(12) $${OD}_{r,min}={ID}_{r}+2t_{r,min}$$

(13) $$D_{r,min}={ID}_{r}+t_{r,mim}$$

(14) $$I_{r}=\frac{\pi }{64}({OD}_{t,min}^{4}-{ID}_{t}^{4})$$

(15) $$Z_{r}=\frac{\pi }{32}\frac{\left({OD}_{r,min}^{4}-{ID}_{r}^{4}\right)}{{OD}_{r,min}}$$

(16) $$A_{i}=\frac{\pi }{4}{ID}^{2}$$

where

*t*—wall thickness, expressed in mm;

*t_r,min_*—minimum reinforced pipe wall thickness, expressed in mm;

*t_l_*—internal liner thickness of the pipe wall, expressed in mm;

*t_s_*—outer sheath thickness of the pipe wall, expressed in mm;

*OD*—outside diameter, expressed in mm;

*ID*—inside diameter, expressed in mm;

*ID_r_*—inside diameter of the reinforced pipe wall, expressed in mm;

*OD_r,min_*—minimum outside diameter of the reinforced pipe wall, expressed in mm;

*D_r,min_*—mean diameter of the minimum reinforced pipe wall, expressed in mm;

*t_r_*—nominal reinforced pipe wall thickness, expressed in mm;

*A_i_*—internal area of the pipe, expressed in square mm^2^;

*A_r_*—minimum reinforced pipe wall cross-section, expressed in mm^2^;

*I_r_*—minimum reinforced pipe walls moment of inertia, expressed in mm^4^;

*Z_r_*—minimum reinforced pipe walls section modulus, expressed in mm^3^;

*M_p_*—pipe mass per unit length, expressed in kg per meter;

*ρ_p_*—pipe mass density, expressed in kg per m^3^.

In [106], a comprehensive analysis is presented, which examines the methodologies employed by different standards when it comes to computing stresses under diverse loading scenarios. At the same time, [106] offers an in-depth comparison of various standards that are currently accessible to designers of fiber-reinforced plastic (FRP) piping systems. It meticulously explores how each of these standards tackles the calculation of stresses arising from different loading conditions.

ASME B31.3 is a code published by the American Society of Mechanical Engineers (ASME) that provides guidelines for the design, construction, and maintenance of process piping systems. The sections 302.3.5(a) and 304.1.2 refer to specific requirements within ASME B31.3.

302.3.5(a): This section likely pertains to the allowable stress values for materials used in process piping. In ASME B31.3, different materials have specific allowable stress values that are used in the design of piping systems. The values can vary depending on factors such as temperature, pressure, and material type. Subsection (a) of Section 302.3.5 may specify how to determine or apply these allowable stress values under certain conditions.

304.1.2: This section is likely in reference to the design temperature limitations for materials used in process piping. ASME B31.3 provides guidelines for selecting materials based on the temperature at which the piping system will operate. Section 304.1.2 [23] may provide information on how to determine the design temperature for a particular application or specify limitations on the use of certain materials at high or low temperatures.

Taking into account references [101,106], Table 13 presents the calculation expressions for wall thickness, comparatively, for pipes made from metallic materials and FRP (fiber-reinforced plastic) materials. In Table 13, the calculation expressions for wall thickness are depicted for both metallic and FRP pipes, allowing for a side-by-side comparison of these materials.

ASME RTP-1 [107] (Reinforced Thermoset Plastic Corrosion-Resistant Equipment) is a standard published by the American Society of Mechanical Engineers (ASME) that focuses on the design, fabrication, inspection, and testing of fiberglass-reinforced plastic (FRP) vessels and equipment used for corrosive and hazardous chemical processes. The standard provides guidelines for manufacturing high-quality FRP equipment to ensure their safety and reliability in various industrial applications. The reference “3A-210” appears to be a section or specific clause within [73], and the calculation expressions for pipe wall thickness can be found in Table 14.

Reference [108] provides guidelines and requirements for the design, fabrication, inspection, testing, and certification of fiber-reinforced plastic (FRP) pressure vessels. According to [106,108], the calculation expressions for pipe wall thickness is:

(17) $$t_{2}=\frac{P\cdot R}{0.001\cdot E_{2}-0.6\cdot P}$$

where t_2_ denotes the structural wall thickness for circumferential stress, P denotes the internal pressure, R denotes the inside radius, and E_2_ denotes the tensile modulus in the circumferential direction.

BS 7159:1989 [109] is a British standard titled “Code of practice for the design and construction of glass-reinforced plastics (GRP) tanks and vessels for use above ground”. This standard provides guidelines for the design and construction of glass-reinforced plastic (GRP) tanks and vessels that are intended for use above ground in various industries, and the calculation expressions for pipe wall thickness is

(18) $$t_{d}=\frac{D_{i}{\cdot P}_{d}}{20\cdot E_{lam\cdot }\varepsilon_{d}-P_{d}}$$

where t_d_ denotes the design thickness of the reference laminate excluding any corrosion barrier (mm), P_d_ denotes the internal design gage pressure (bar), D_i_ denotes the internal diameter (mm), E_lam_ denotes the modulus of elasticity of the laminate (MPa), and ε_d_ denotes the design strain, typically 0.0009–0.0018.

ISO 14692 [57] is an internationally recognized standard that serves as a comprehensive guideline and set of requirements for the design and installation of glass-reinforced plastic (GRP) piping systems. These systems are extensively utilized in a wide range of industrial applications, where they play a vital role in the conveyance of various fluids. The overarching objective of ISO 14692 is to establish a framework that guarantees the safe and dependable operation of GRP piping systems. According to [65,66], pt. 7.1 and pt. 8.3 the wall thickness is:

(19) $$t=\frac{P_{qs}\cdot D}{2\cdot 10\cdot \sigma_{qs}}$$

(20) $$P_{qs}=A_{1}\cdot A_{2}\cdot A_{3}\cdot P_{q}$$

(21) $$P_{q}=f_{1}\cdot LTHP$$

The following definitions are provided: *t*—reinforced wall thickness (mm); *P_qs_*—service qualified pressure (bar); *D*—mean diameter of the reinforced wall, i.e., (*2R_i_ + t*); *R_i_*—inside radius of the reinforced wall (mm); *F_qs_*—qualified service stress (MPa); *A*_1_—partial factor for temperature; *A*_2_—partial factor for chemical resistance; *A*_3_—partial factor for cyclic service; *P_q_*—qualified pressure (bar); *f*_1_—part factor equivalent to 97.5% confidence limit of the *LTHP*, default = 0.85; and *LTHP*—Long-term Hoop Pressure (bar).

The above formulas, in the context of fiber-reinforced plastic (FRP) pipe design, deal with the critical aspect of sustained loads arising from internal pressure. This scenario gives rise to circumferential stresses within the pipes. Engineers and designers use a strength criterion to calculate the required wall thickness values for FRP pipes, ensuring they can withstand these internal pressures safely.

Comparatively, we also examine the scenario of sustained loads resulting from external pressure, which is equally important in the design process. In this case, engineers determine the maximum allowable external pressure for known wall thickness values (Table 15 and Table 16). This analysis ensures that the FRP pipes can endure external forces, such as those imposed by soil, water, or other external factors.

These analyses are essential to ensure the structural integrity and safety of FRP pipes in various industrial applications. Designing pipes that can withstand both internal and external pressures is a critical engineering consideration, and adherence to applicable design codes is fundamental toachieving this objective.

The stress components in the case of FRP (fiber-reinforced plastic) pipes are presented below.

The total circumferential (hoop) stress in pipes or vessels has two fundamental components: one attributed to the internal pressure ($\sigma_{h,p})$ and another resulting from external loading ($\sigma_{h,a})$, particularly in the case of buried pipes. These stress components must be carefully assessed, calculated, and considered during the design and analysis processes to ensure the structural integrity and safety of the component in its operational environment.

(22) $$\sigma_{h,total}=\sigma_{h,p}+\sigma_{h,a}$$

The expression for circumferential (hoop) stress in the case of FRP (fiber-reinforced plastic, subjected to internal pressure) pipes is

(23) $$\sigma_{h,p}=\frac{pD_{r,min}}{2t_{r,min}}$$

The expression for circumferential (hoop) stress in the case of FRP (fiber-reinforced plastic) pipes subjected to external loads (as in the case of buried pipes) is

(24) $$\sigma_{h,a}=r_{c}D_{f}E_{hb}\frac{∆y}{D_{r,min}}\frac{t_{r,min}}{D_{r,min}}$$

where $\frac{∆y}{D_{r,min}}$ is the predicted vertical pipe deflection for a buried FRP (fiber-reinforced plastic) and is calculated according to AWWA Manual M45(second edition), $r_{c}$ is the resounding coefficient and depends on the value of the internal pressure, and $E_{hb}$ is the hoop bending modulus(MPa).

The axial stress ($\sigma_{a,total})$ has the following components: from internal pressure ($\sigma_{a,p})$, from bending ($\sigma_{a,i})$, from temperature ($\sigma_{a,t}$), from compression ($\sigma_{a,c}),$ and from pressure trust ($\sigma_{a,f})$:

(25) $$\sigma_{a,total}=\sigma_{a,p}+\sigma_{a,i}+\sigma_{a,f}+\sigma_{a,c}+\sigma_{a,t}$$

The axial stress from the “pressure thrust” phenomenon represents a component of stress that occurs in cylindrical structures or pipes, especially when they are subjected to internal pressure. This axial stress ($\sigma_{a,f})$ is caused by the internal pressure acting on the interior surface of the structure and tends to push or extend the cylinder in the axial direction.

The key aspect of axial stress from the “pressure thrust” phenomenon is that it can generate a significant force that tends to push or thrust the component at the end of the structure, depending on how it is anchored. This force can be substantial and needs to be considered in the design and analysis of cylindrical structures or pipes to ensure their safety and stability.

It is important to note that the axial stress from the “pressure thrust” phenomenon ($\sigma_{a,f})$ is a specific component of the stress exerted by internal pressure on cylindrical structures and can be distinct from other types of stresses, such as circumferential stress.

The axial stressfrom internal pressure is the following:

(26) $$\sigma_{a,p}=\frac{p\cdot D_{r,min}}{4\cdot t_{r,min}}$$

For an unrestrained FRP pipe, the axial stress from internal pressure is the following:

(27) $$\sigma_{a,p}=\nu_{ah}\frac{p\cdot D_{r,min}}{2\cdot t_{r,min}}$$

The longitudinal stress due to bending ($\sigma_{a,i})$ in the case of fiber-reinforced plastic (FRP) pipes depends on the stress intensification factors in-plane (${SIF}_{ai})$ and out-of-plane (${SIF}_{ao})$.

Longitudinal stress due to bending occurs when an external load or moment is applied to an FRP pipe, causing it to bend or deform. This type of stress acts parallel to the length (longitudinal direction) of the pipe.

Several factors influence the longitudinal stress in bending, including the magnitude and distribution of the applied bending moment, the pipe’s geometry, and, most importantly, the material properties of the FRP. The stress intensification factor (SIF) is a dimensionless parameter used to quantify how the stress in a specific location or direction differs from the nominal or uniaxial stress. It accounts for the complex stress distribution in structures subjected to bending, torsion, or other loading conditions.

In the context of FRP pipes under bending, the in-plane ${SIF}_{ai}$ accounts for the intensification of stress within the plane of the bend. It considers how the FRP material responds to bending forces applied within the plane of the pipe’s curvature.

The out-of-plane ${SIF}_{ao}$, on the other hand, deals with the intensification of stress perpendicular to the plane of bending. It addresses how the FRP material reacts to forces acting out of the plane of curvature.

The calculation of longitudinal stress in FRP pipes under bending involves considering the in-plane and out-of-plane SIFs. These factors modify the nominal or uniaxial stress experienced by the material due to bending, and they depend on the pipe’s geometry and the specific loading conditions. The SIFs are influenced by the anisotropic nature of FRP materials, meaning that the material properties vary with direction. Understanding how these properties interact with the SIFs is crucial for accurately assessing the longitudinal stress.

(28) $$\sigma_{a,i}=1000\cdot \frac{\sqrt{{\left({SIF}_{ai}\cdot M_{i}\right)}^{2}+{\left({SIF}_{ao}\cdot M_{o}\right)}^{2}}}{Z_{r}}$$

(29) $$Z_{r}=\frac{\pi }{32}\frac{\left({OD}_{r,min}^{4}-{ID}_{r}^{4}\right)}{{OD}_{r,min}}$$

(30) $$\sigma_{af}=\frac{F_{a}}{\frac{\pi }{4}\left({OD}_{r,min}^{2}-{ID}_{r}^{2}\right)}$$

(31) $$\sigma_{ac}=E_{a}\cdot \frac{OD_{r,min}}{2\cdot C\cdot 1000}$$

(32) $$\sigma_{at}=\alpha_{a}\cdot \left(T_{install}-T_{design}\right)\cdot E_{a}$$

(33) $$\sigma_{a,total}=\frac{F_{a}}{A_{r}}+\frac{\sqrt{{\left({SIF}_{ai}\cdot M_{i}\right)}^{2}+{\left({SIF}_{ao}\cdot M_{o}\right)}^{2}}}{Z_{r}}$$

where $\sigma_{a,i}$ is the axial stress from bending; $\sigma_{a,t}$ is the axial stress from temperature; $\sigma_{a,c}$ is the axial stress from compression; $\sigma_{a,f}$ is the axial from pressure trust; $Z_{r}$ is the modulus of the cross-sectional resistance of the pipe; $M_{i}$ is the in-plane bending moment; $M_{o}$ is the out-of-plane bending moment; *ID_r_* is the inside diameter of the reinforced pipe wall; *OD_r,min_* is the minimum outside diameter of the reinforced pipe wall; $A_{r}$ is the cross-sectional area of the pipe; $T_{install}$ is the installation temperature; $T_{design}$ is the design temperature; C is the compressibility factor that depends on the manufacturer; $\alpha_{a}$ is the coefficient of thermal expansion of the material; $E_{a}$ is the axial modulus of elasticity; and $F_{a}$ is the axial component of the force acting on the pipe.

## 6. The Behavior of FRPs in Different Environments

The most important properties of reinforced polymers are corrosion resistance, durability, strength degradation (tensile strength), and long-term resistance.

The main factors that influence corrosion resistance are the environment, temperature, loading conditions, and concrete cover thickness.

The durability of the reinforced polymer is also influenced by the environmental conditions, temperature, resin types, and surface treatments, and it is strongly dependent on the humidity, which is influenced by the water–cement ratio, the concrete cover thickness, and the immersion environment.

Some of the most popular FRP rebar applications are bridges, barrier walls, and decks. These specific types of structures require materials that are non-corrosive and long-lasting, which are both features of FRP rebar.

A glass-fiber-reinforced polymer (GFRP), also known as glass-fiber-reinforced plastic, is a composite material in the composition of which there is a braiding consisting of fiberglass and polyester. Due to their non-corrosive features, GRRPs are successfully used for boat construction and architectural applications.

Basalt-fiber-reinforced polymers (BFRPs) can be used in diverse applications, from concrete for roads, bridges, and airport runways to dams and other projects. One of the most important characteristics of this basalt fiber is that it can have a controllable thermal conductivity, depending on its density and fineness. And this feature makes it useful in applications where it acts as a thermal-insulating composite material.

In conclusion, these fiber-reinforced polymers will interact with many kinds of environments: tap water, seawater, deionized water, concrete, and alkaline solutions (laboratory or outdoor conditions).

Different studies were found in the literature in which these reinforced polymers were tested (corrosion resistance, durability, tensile strength) under the following conditions (temperature, environment, etc.).

The mechanical properties of FPR are affected by the alkaline environment.

Seawater has also been observed to have a similar effect to tap water on the degradation mechanism of FRP bars, but seawater salt inhibits the diffusion process of water in the matrix. In addition, on the surface of FPR bars immersed in seawater, a thin layer of salt was observed, especially when exposed to high-temperature conditions. Hence, the durability of FRP bars in seawater is superior to those in tap water at higher temperatures [110]. The mechanical properties of FRP bars are affected by temperature, especially in the range of 20 °C to 80 °C.

Because extreme temperatures influence the properties of FPRs, there are research studies investigating their mechanical properties in high-temperature and extremely cold environments.

For instance, Wang et al. [111] investigated the mechanical properties of GFRP bars at temperatures ranging from 20 to 600 °C. A temperature of 350 °C was found to be a critical temperature from a mechanical properties point of view because the bars lost about half of their tensile strength. Below this temperature, the elastic modulus of the bars remains 90%. It has been observed that the mechanical properties of the FRP bar deteriorate more slowly with increasing temperature, while for the GFRP bar, degradation is much more sensitive to temperature rise. These results are complemented by those of Ashrafi et al. [112], which additionally demonstrate that in addition to temperature, bar diameter, fiber type, and resin type influence their properties. It can be concluded that the mechanical properties of FRP bars gradually decrease with increasing temperature, and at high temperatures, they undergo rapid deterioration. At temperatures above 300 °C, traction properties decrease rapidly.

In dry conditions, FRP bars have excellent durability compared to indoor conditions in field exposure experiments, but they degrade quickly in wet environments. The mechanical characteristics and durability of FRP bars are influenced by both external environmental factors and internal parameters. FRP bar failure is most likely caused by the corrosion of the alkaline solution. FRP bars in seawater and tap water experience deterioration due to hydrolysis.

Robert and Benmokrane [113] reported that exposure to alkaline solutions has a greater impact on the durability of GFRP bars than exposure to moist mortar. Chen [114] tested GFRP bars embedded in concrete with a water-to-cement ratio of 0.55. These specimens experienced a 10% and 39% decrease in tensile strength after being exposed to wet and dry cycles and an alkaline solution (pH of 12.7) for 90 days at 40 °C.

According to EI-Hassan et al. [115], the performance of the GFRP reinforcing bars was directly affected by the saline environment.

In an alkali solution at 60 °C for 5000 h, GFRP bars soaked in epoxy and vinyl ester resins with a higher retention rate than polyester resin substrates, according to another study [116].

The interfacial adhesion between epoxy and vinyl ester resins and glass fibers is greater for epoxy- and vinyl ester-based GFRP bars than polyester-based GFRP bars, leading to better mechanical properties. Compared to GFRP bars exposed to alkaline solutions at 20, 40, and 80 °C, the tensile strength retention rate of modified vinyl ester resin GFRP bars is better [117].

Glass/vinyl ester FRP bars have the highest physical and mechanical properties and the lowest degradation rate after being conditioned in an alkaline solution, while Basalt/vinyl ester FRP bars have the lowest physical and mechanical properties and a significant degradation rate [118].

Reinforcing materials can have a significant impact on their mechanical properties and durability due to various fibers, surface treatments, and matrices. Enhancing the durability of FRP bars requires modification of resin to improve weather resistance. Modified acrylic resin is commonly utilized in the interior decoration of cars, for instance.

The mechanical properties of the GFRP bars based on the modified acrylic resin are maintained, and the tensile properties of the resin stay over 90% after being immersed in an alkaline solution at 60 °C [119].

Moreover, they can offer more safety in handling and also can raise the evacuation time in case of a fire event. Many studies compared the durability of FRP bars covered in concrete in marine water and tap water environments. GFRP bars wrapped in concrete show a retention speed higher in marine water than tap water [120,121], the results of which are similar to the results obtained for simple bars. Another study [122] obtained the opposite effect. The authors specified that sodium chloride from marine water entered these concrete pores and modified the internal molecular composition, leading to an increase in OH^−^, so the alkalinity increased, with a negative impact on traction behavior. A few studies’ investigations have been carried out regarding the durability of BFRP bars covered in concrete.

Usually, OH^−^ bonds interact with the resin, damaging the molecular chain and eventually dissolving it. The corrosive ions and water molecules quickly enter the matrix fiber interface through weak cracks, causing faster aging and strength reduction. Normally, the seawater is plentiful in Cl^−^ ions that can produce significant corrosion. So, Salloum and colab. [110] reported that the tensile strength retention rate is 11% higher in seawater than for tap water after experiencing 540 diving days at a temperature of 50 °C. Also, the authors concluded that the tensile strength rate after 132 days at 80 °C is 4.7% more than when tap water is used. They noticed the presence of a thin layer of salt on the bar surface that is formed at higher temperatures. Without a doubt, this salt layer will prevent water molecules from entering the FRP bar structures. In conclusion, at higher temperatures, the durability of FRP bars in seawater is, overall, superior to the durability of those presented in tap water. The deterioration of FRP bars in real conditions can be well studied by exposure to the field in which they are used daily.

Lu and colab. [123] tested the FRP bars covered in concrete in outdoor conditions in seawater at temperatures between 0.9 and 24 °C. The authors discovered that after one year, there was a reduction of strength of almost 35% and 11%, respectively, for wrapped and unwrapped BFRPs.

After a period of 18 months, the authors concluded that the tensile strength of empty FRP does not change in conditions of high heat and humidity. Indeed, 98.1% and 97.6% were the results obtained for the tensile strength retention rate of GFRPs covered in concrete.

Benmokrane and colab. [124] reveal that the bars that are based on carbon fibers present a higher resistance to alkaline corrosion. Unfortunately, the tensile strength retention was lower for 25 mm bars than for 5 mm bars when the authors soaked the bars in an alkaline solution because it seems that a failure error correlated with the delay effect of shear.

Also, Lu and colab. [123] studied the durability of covered and empty bars in a seawater environmentat 60 °C for one year. The authors concluded that a covered bar is liable to become corroded because of the alkalinity of concrete [125]. Secondarily, a reaction between silicon dioxide and the alkali from concrete appears [126]. It was also observed that when we are talking about the presence of a coral aggregate, rapid degradation occursbecause of the coral pores that can easily suck the water from the environment.

The tensile strength rate of BFRP was also studied by Rifai and colab. [127] in an alkaline environment, with wet concrete at a temperature between 20 and 60 °C. The authors concluded that the tensile strength rate of BFRP bars tested for 9 months of sinking presented noticeable losses at temperatures of 50 °C, and no effect was observed at room temperature. In a strange way, the elasticity of BFRP bars has increased by 6.5%, which was probably the result of the resin strengthening. At the end of their research, the authors of this study specified that, at high temperatures, the deterioration appears fast, so the tensile strength rate is lower.

Other authors, like Lu and colab. [125], examined the durability of bars in marine water, which modified the thickness of the protective layer after 360 days of exposure.

So, the alkalinity of the material from which the layer is made will decrease after being submerged in marine water because of the environment. The authors showed that the tensile strength of the 20 mm thick layer is lower than that of the 10 mm covering. In this paper, the authors also discuss the appearance of an alkali equilibrium when the thickness of the layer is 10 mm and the temperature conditions are 40 °C and 60 °C for 180 and 90 days.

Eventually, the mechanism and durability character of FRP bars can be affected by external and internal environmental parameters. The most important effect of the deterioration of FRP is provided by alkaline corrosion. The most common chemical process that appears in the deterioration of FRP bars is hydrolysis. The durability of FRP is a little higher in marine water than in tap water.

Without out a doubt, when we discuss wet concrete covering, we discover a higher tensile strength than in an alkali environment. The main factor is the water–cement ratio, and the main disadvantage remains the alkalinity that appears inside the concrete. Regarding the temperatures, we can discuss different behaviors. Bars have good resistance at a lower temperature range between −100 and 0 °C, but after a temperature of 300 °C, the strength is almost lost. It is also important that freeze–thaw cycles do not occur, and also, long-period humidity exposures should be avoided. The connection between the transaction load and the environment is also important. When this is higher, it is normal for the mechanical proprieties to drop because, in all the studies, the humidity factors created problems, and it is mentioned several times that the best way to protect the bars is to use a dry environment as much as possible. All these environmental behaviors definitely affect the resistance of bars as well as other factors.

The alkali environment stands out because, unfortunately, the deterioration of the bars is very fast in this kind of environment. Finally, regarding the environmental behavior from all current studies, the FRP bar manufactured with vinyl ester presents a higher capacity performance than those with epoxy and polyester resins.

All the studies have shown that the rate of degradation of GFRP and BFRP bars varies based on an order of alkali solution > water environment > acid solution > salt solution. Also, GFRP bars exhibited superior corrosion resistance than BFRP bars in the alkali, acid, and salt solutions, while the corrosion resistance in the water solution was lower.

## 7. The Use of Fiber-Reinforced Polymeric Composites in Pipelines

Corrosion of steel piping systems is a major problem in the offshore and onshore petroleum gas industry. Leakage of petroleum fluids pollutes the environment and could lead to explosions. Repairing or replacing corroded equipment involves high costs. Fiber-reinforced polymer composites, and especially glass-fiber-reinforced plastic (GFRP),are used to enhance the corrosion resistance of the piping systems as coatings or in the construction of pipes. GFRP pipes are composite pipes made with glass fibers and thermoset resins, such as unsaturated polyester or vinyl ester. These pipes are manufactured through centrifugal casting and/or filament winding of glass fibers [128,129].

The durability of GFRP pipes has been a cause of concern and a difficult task because of the extensive mechanical tests on the components. The hydrostatic and cyclic pressure, uniaxial tensile and compressive, bending, and combined loading tests are often utilized to ascertain their application in the oil and gas industry with fair durability and safety [129].

The chemical resistance, service temperature, and mechanical properties of GFRP depend on working fluids but also on resin, additives, fiber type and the % used, dimensions and angle of structural reinforcing fillers, reinforcement materials, matrix materials, the technology of manufacturing, surface preparation, etc. [130,131,132].

Glass-reinforced epoxy (GRE) pipes have superior axial and circumferential mechanical values compared to other glass-fiber-reinforced piping systems [96]. They exhibit excellent chemical resistance against hydrocarbons, seawater, and mild corrosive liquids. The most used resins for GRE pipes are DER 330 and EPIKOTE 827/8281 [133].

API 15HR [134] presents the availability of safe, dimensionally and functionally interchangeable high-pressure fiberglass line pipes with pressure ratings from 3.45 MPa to 34.5 MPa, with 1.72 MPa increments for pipes ≤ NPS 12 inches and 0.69 MPa increments for pipes ˃ NPS 12 inches. This specification is limited to mechanical connections, and the technical content provides requirements for performance, design, materials, tests and inspections, marking, handling, storing, and shipping. Critical components are items of equipment with requirements specified in this document. This specification is applicable to rigid pipe components made from thermosetting resins and reinforced with glass fibers. Typical thermosetting resins are epoxy, polyester, vinyl ester, and phenolic resins. Thermoplastic resins are excluded from the scope of this specification. Any internal liners applied shall also be made from thermosetting resins. Fiberglass line pipe for use in low-pressure systems is covered in API SPEC 15LR [102], which also covers filament wound (FW) and centrifugally cast (CC) fiberglass line pipe and fittings for pipes with diameters up to and including 24 in. in diameter and up to and including 1000 psig cyclic operating pressures. In addition, according to the manufacturer’s choice, the pipe may also be rated for static operating pressures up to 1000 psig. It is recommended that the pipe and fittings be purchased based on the cyclic pressure rating. The standard pressure ratings range from 150 psig to 300 psig in 50 psig increment and from 300 psig to 1000 psig in 100 psig increments, based on either cyclic pressure or static pressure. Quality control tests, hydrostatic mill tests, dimensions, weights, material properties, physical properties, and minimum performance requirements are included [8]. Also, ASTM C582-23 [70] covers composition, thickness, fabricating procedures, and physical property requirements for glass-fiber-reinforced thermoset polyester, vinyl ester, or other qualified thermosetting resin laminates comprising the materials of construction for RTP corrosion-resistant tanks, piping, and equipment. This specification is limited to fabrication by contact molding. Laminates shall be classified according to type, class, and grade: Types I and II; Classes P and V. Tensile strength and tangent modulus of elasticity, flexural strength, glass content, thickness, hardness, chemical resistance, and surface flame-spread classification tests shall be performed to conform to the specified requirements.

There are four major materials utilized in order to produce fibers for the industry: carbon, glass, aramid, and basalt, to form CFRP, GFRP, AFRP, and BFRP, respectively [29,31,135].

The constituent materials, along with the processes by which they are combined, determine the properties of a finished composite part. E-glass is the most widely used reinforcing fiber. Silica sand, limestone, and other minerals are melted in a furnace and allowed to fall through tiny holes in a platinum plate to create fibers around 5–24 μm in diameter [28].

The most used resin in glass fiber composites (GFRP) is unsaturated polyester. Vinyl esters are tougher and more water-resistant than polyester. Epoxy resins outperform most other resins and are usually used with carbon fiber. Phenolic resins have lower mechanical properties but very good fire resistance. Several other resin systems also exist.Mineral fillers such as calcium carbonate are often included to reduce cost, as less resin is needed while improving other properties. Other additives can be included, e.g., fire retardants, UV absorbers, or toughening agents. Fibers can be incorporated directly in some processes but more often are converted into fabrics that may be uni-/bi-/multi-axial, woven, knitted, braided, needle-punched, or simply chopped and bound into a random-oriented fabric. Textile engineering is an important aspect of optimizing composite manufacturing, and 3D preforms are increasingly being used to align fibers exactly where they will provide the best properties [132].

In some manufacturing processes, fibers are pre-impregnated with resin, which usually needs to be kept in a freezer to stop the resin polymerization before the time. Bulk molding compound (BMC) is a mixture of short, chopped fibers, resin, and filler used in injection and compression molding. It is also called dough molding compound (DMC). Sheet molding compound (SMC) is made by chopping longer (25 mm+) fibers into a layer of resin [95].Reinforcing and thermoplastic fibers can be commingled to make a fabric, which can be referred to as a raw thermoplastic material. When heated, the thermoplastic fibers melt to form a matrix. Also, short fibers can be ‘compounded’ with thermoplastic resin for injection molding. Commonly used core materials may be polymer foams such as polyurethane, PVC, acrylic, or honeycomb structures made from aluminum, paper, Nomex^®^(a Kevlar^®^-based paper), or other polymers. Balsa wood is an excellent natural core material with good fire resistance.

Adhesive bonding is probably the most versatile joining technology available to the engineer, and in the case of composites, it is often the most practical way to combine them with other materials, such as metals and polymers. Indeed, composites themselves can be described as a product of adhesion between a resin (thermoplastic or thermoset) and the structural fibers within. However, in the vast majority of cases, adhesive bonding should be considered a pseudo-two-dimensional, surface-driven process where stresses and strains are transferred across an interface between two planes:the substrate (often referred to as the adherend) and the adhesive. Where the adhesive bond thickness is low, i.e., less than 100 µm, the adhesive could almost be described as a single interphase region between two adherends. As the adhesive layer thickens, the adhesive becomes a component, and the bond should be described as a sandwich of two adherends, two interphases, and a layer of adhesive, with the bulk properties of the adhesive playing a larger role in the joint performance. Such an emphasis on load transfer at the interface between the adhesive and the adherend has particular ramifications on composites in particular, due to the material properties of the supporting resin and the interlaminar adhesion between fiber layers in the z-direction. The result of this is often seen by the failure zone moving out of the adhesive/interface region and into the composite material either in the resin-rich surface or between the laminar planes of the first and second ply, where the composite resin becomes the weakest region within the joint. Despite this unique aspect of composites, adhesives, due to their ability to spread loads over large areas with minimal impact on the underlying surface, are often the joining process of choice when compared to mechanical attachment, where there is almost inevitable fiber damage and the creation of regions of high stress [136].

Different joining technologies are available, namely, bonded connections, (un-)restrained O-ring connections, screw connections, and laminated connections for repairs and/or tie-ins. GRE meets the ISO 14692-1,2,3,4 [57,98,99,100] standard, which is regarded as the general standard by piping GRP engineers.

The winding angles of glass-fiber-reinforced plastic (GRP) pipes are typically optimized based on the specific application and desired mechanical properties. The winding angles can affect the mechanical performance of the pipes, including their resistance to internal and external pressures, creep, fatigue, and deformation [137].

In reference [138], Sebaey presents an experimental investigation on the composite pipe’s internal pressure capacity and impact. The paper tested four pipe configurations made with the winding angles of ±45, ±55, ±63, and combined of ±63/±45/±55. All the specimens have the same nominal dimensions of 110 mm internal diameter, 3.8 mm wall thickness, and 448.5 mm length. Internal pressure tests were conducted under a closed-loop control system to ensure safety. After the internal pressure test, low-velocity impacts were applied to the specimens using a drop-weight impact tester. The pressure results showed the superior internal pressure capacity of the specimens manufactured using the winding angles ±55, which is in agreement with the data available in the literature. The specimens made of 63 winding angles and the ones made of the hybrid angles both showed a close value of the pressure capacity, with advantages for the ±63 specimens due to the gaps resulting during the manufacturing of the ±45 layers. The specimens made of all ±45 angles are not recommended for any oil and gas applications based on our current analysis and the manufacturing difficulties we faced with this configuration. From the impact results, the combined orientations ±63/±45/±55 pipe can be considered as a competitor to the ±55 pipes. Although, their low-pressure capacity might limit their usage in low-pressure applications. The pipes manufactured using the ±63 orientations show very low damage resistance, as assessed using all the prescribed parameters. Obviously, the decision is clear in the case of selecting a pipe based only on the pressure capacity. However, pipes are not only subjected to internal pressure, and they might suffer from impact, bending, axial loadings, aging, etc., during their lifetimes. The response of composite parts under any of these conditions is highly dependent on the winding angles. For this reason, a recommendation can be drawn to design composite pipes based on the satisfaction of not only the internal pressure but also the other expected loading and working conditions.

Wang et al. in [139] conducted a study on reinforced thermoplastic pipes (RTPs) in order to optimize the multiple winding angles of RTPs under internal and external pressures. The study used three-dimensional (3D) thick-walled cylinder theory with the 3D Hashin failure criterion and theory of the evolution of damage to composite materials to analyze the progressive failure of RTPs. The model was verified by experiments, and a multi-island genetic algorithm was used to establish an optimal scheme for winding angles capable of withstanding maximum internal/external pressure. Another study by Baali et al. [137] focused on wound glass-fiber-reinforced polymer (GRP) pipes and their mechanical characterization. The study aimed to optimize the performance/cost ratio of these materials by designing and manufacturing orthotropic filament-wound GRP pipes with different fiber orientations, such as ±45°, ±55°, and ±70°. Experiments were conducted on GRP pipes wound at different angles, and the results were compared with analytical methods.These studies demonstrate that the winding angles of GRP pipes can be optimized to enhance their mechanical performance. However, it is important to note that the specific winding angles used in practice may vary depending on factors such as pipe diameter, application requirements, and manufacturing processes.

The percentage of fiber in glass-reinforced plastic (GRP) can vary depending on the specific application and desired mechanical properties. The most common type of glass fiber used in GRP is E-glass, which is alumino-borosilicate glass with less than 1% alkali oxides. The fiber content in GRP can range from approximately 15% to 60% by weight [140]. It is important to note that the fiber content is just one factor that contributes to the overall properties of GRP. Other factors, such as the type of resin used and the manufacturing process, also play a significant role in determining the material’s characteristics [141].

The evaluation of the mechanical properties and chemical resistance of these pipes is essential to assess their long-term performance in specific applications. The results of a study by Hadrami et al. in reference [105] evaluate the chemical resistance and mechanical properties of GFRP pipes for application in oil and gas plants. Pipe specimens were preconditioned by filling them with 7.5% HCl, 8% H_2_SO_4_, and a mixture of 2% NaOH and 2% KMnO_4_ solutions maintained at 63 °C. The preconditioned and fresh pipe specimens were exposed to clean and oily water (a mixture of 10% toluene, 10% kerosene, and 80% brine water (30 g/L NaCl)) maintained at 93 °C and 15 bar pressure for 1000 h. The fresh, preconditioned, and exposed pipe specimens were tested to evaluate their axial and hoop tensile strength, water absorption, and loss upon ignition. The experimental results did not show a major change in the properties of evaluated pipes owing to exposure to clean and oily water at high temperatures and pressures. There was minimal or no loss in the axial and hoop strength of the preconditioned or exposed pipes. The absorption and loss of ignition did not increase significantly, both due to pre-conditioning and evaluated exposure conditions. The results indicate that GFRP pipes are suitable for application in the oil and gas industry.

## 8. Conclusions

FRP composites represent a versatile and indispensable class of materials with a wide range of applications across various industries. Their adaptability and diverse classification criteria allow customized solutions to meet specific needs, from civil engineering and aerospace to automotive, marine, and more. FRP composites are characterized by the type of reinforcing fiber, the matrix material, the manufacturing process, fiber orientation, and the intended application. These classification options provide a flexible framework for engineers to select the most suitable materials and manufacturing methods.

The manufacturing technologies for FRP composites provide a versatile range of methods, allowing them to serve a wide spectrum of applications, from automotive and aerospace components to construction materials and biomedical devices. Material efficiency is a focal point for many automated and advanced manufacturing methods, addressing environmental concerns and resource efficiency by minimizing material waste and reducing energy consumption compared to traditional processes.

Automated and advanced manufacturing technologies provide precision and quality control, ensuring consistent and reliable composite products. This level of precision is crucial, particularly in industries like aerospace and automotive manufacturing, where safety and structural integrity are essential.

The choice of manufacturing technology is influenced by various factors, including production volume, initial investment, and labor costs. While advanced techniques offer superior quality and precision, they often require substantial investments in specialized equipment, potentially limiting their accessibility for smaller manufacturers.

The FRP manufacturing sector is marked by a constant evolution in innovation to refine existing methods and develop new approaches. The innovation primarily centers on the enhancement of energy efficiency, waste reduction, and the optimization of material properties.

Selecting the most appropriate fabrication method should be guided by a comprehensive assessment of the unique needs of the project, taking into account elements such as cost, complexity, production volume, and the desired attributes of the final product.

Fiber-reinforced polymer (FRP) materials have gained significant importance in various engineering applications, particularly in the design and fabrication of structural components, such as pipes, pressure vessels, and aerospace components. One of the key parameters that engineers often focus on in the design process is the wall thickness of these FRP components.

The selection of an appropriate wall thickness is a critical aspect of the mechanical design of FRP structures. This choice is influenced by a variety of factors, including the desired mechanical properties, the intended application, environmental conditions, and regulatory requirements. The directional stiffness and strength characteristics of FRP materials make them particularly well-suited for applications where these properties play a pivotal role in the performance of the structure.

Directional stiffness refers to the ability of the FRP material to resist deformation when subjected to loads in specific directions. FRP composites can be engineered to have anisotropic properties, meaning they exhibit varying stiffness and strength in different directions. This anisotropy is achieved by controlling the orientation of the reinforcing fibers within the polymer matrix during the manufacturing process. Engineers carefully design and analyze the laminate lay-up to achieve the desired directional stiffness tailored to the specific load conditions that the structure will encounter.

Strength is another crucial consideration in the design of FRP components. The tensile, compressive, and shear strength of FRP materials are key factors in determining the wall thickness required to ensure structural integrity and safety. The direction of the applied loads plays a critical role in determining the stresses within the structure. Engineers need to account for these directional stresses when designing FRP components to ensure that they can withstand the expected loads and operate safely within their intended service life.

A comprehensive evaluation of material properties in AFRP, BFRP, CFRP, and GFRP aids in informed material selection for diverse engineering contexts.In terms of tensile strength, CFRP’s lower weight and higher tensile stress enable significant weight reduction, while BFRP excels in this regard. GFRP further enhances tensile strength, especially in hybrid forms. Compressive strength varies, with CFRP showcasing the highest, influenced by factors like basalt fibers and matrix choice. Design standards guide FRP shear strength, considering moisture effects in epoxy and the stiffness of FRP rebars.In summary, FRP composites offer tailored solutions, balancing factors like tensile and compressive strengths, shear behavior, and adherence to standards in applications ranging from structural components to GRP piping systems. Understanding these properties is essential for effective material selection and engineering design.

Studies on the durability of FRP-steel joints reveal that exposure to environmental conditions, including humidity, seawater, high/low temperature, and ultraviolet (UV), can degrade the properties of the bond. Hence, the durability of steel-FRP joints in different environmental conditions can play a key role in the performance of in-service structures.

The FRP bars were very quickly degraded in the alkali solution, followed by the water environment, the acid solution, and the salt solution. GFRP bars showed better corrosion resistance than BFRP bars in the alkali, acid, and salt solutions, while the opposite was found in the water solution.

The combination of humidity with other factors, like salt and temperature, accelerates the deterioration level. Exposing a joint to a marine environment can double the reduction in strength and stiffness compared to being exposed to 90% humidity. Hydrothermal conditions are recognized as the most deteriorative environment.

Exposure to the natural environment provides more reliable results and can be used to validate the laboratory results, which may underestimate the bond durability due to the accelerated procedure.

## Figures and Tables

**Table 1:** Types of glass fibers.

Glass Fiber Type	Characteristics/Advantages
E-glass	High strength and electrical resistivity
A-glass	High durability, strength, and electrical resistivity
S-glass	High tensile strength
C-class	High corrosion resistance
D-glass	Low dielectric constant
R-glass	High strength and acid corrosion resistance
AR-glass	High alkali resistance

**Table 2:** Types of Kevlar fibers.

Kevlar Fibers Type	Characteristics/Advantages
K-29	Regular, light weight, medium thermal and electrical resistance
K-49	High modulus, light weight, good thermal and electrical resistance, traction resistance
K-100	Colored, light weight
K-119	High durability, higher elongation, flexible and more fatigue resistant, light weight, good traction resistance
K-129	High strength, higher tenacity, high thermal and electrical resistance, light weight, good traction resistance
K-149	Ultra-high modulus, highest tenacity, light weight, high thermal and electrical resistance, high traction resistance

**Table 3:** Description of FRP manufacturing technologies.

Process Name	Process Description	Application	Advantages	Disadvantages
Hand lay-up	It is the simplest and most extensively employed open-mold composite manufacturing method. In this process, fiber preforms are initially positioned within a mold, and a thin layer of an antiadhesive coating is applied to facilitate easy removal. Subsequently, resin material is poured or brushed onto the reinforcement material, and a roller is used to effectively drive the resin into the fabric to enhance interaction between successive layers of the reinforcement and the matrix materials [18].	Manufacturing large structures in low quantities in the marine sector (boat hulls and related components), automotive industry (such as car body panels), energy field (wind turbine blades), transportation (including large containers), and household applications (swimming pools, bathtubs, etc.) [17].	Low-cost equipment and operation. Suitable for large components. Adaptability to complex shapes. Environmental sustainability (involves lower energy consumption and fewer emissions compared to some automated processes).	Due to the open-mold, the products have a single well-finished surface, necessitating a secondary trimming operation [17]. Labor-cost heavy and inefficient [23].
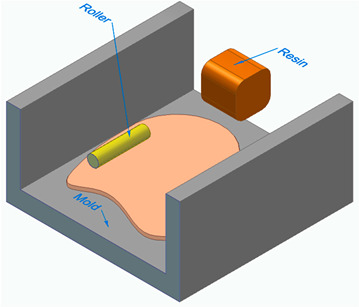 (Adapted form [20]).				
Spray lay-up	The spray-up technique shares similarities with hand lay-up, with the main distinction being the utilization of a spray gun that dispenses resin and chopped fibers onto a mold [18].	The method is suitable for low and moderate production of large FRP composite structures, such as bathroom units and ventilation hoods [17].	Simplicity. Faster FRP production and greater shape complexity compared to low-cost hand lay-up technique equipment.	The parts have only one finished part surface. Resin waste. Reduced fiber orientation control. Air entrapment.
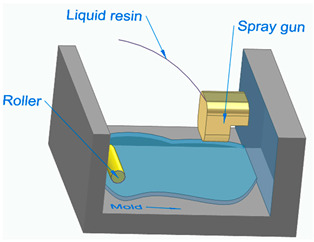 (Adapted form [20]).				
Pultrusion	This process involves the continuous pulling or reinforcing of fibers through a resin bath, followed by a shaping die or heated die that cures the composite material.	FRP bars, profiles, and strips [11].	This method is simple, effective, and adaptable and represents the most economical solution for producing continuous fiber structural composites characterized by a constant cross-sectional profile [19]. It is suitable for mass production.	It is less suitable for complex or intricate shapes and components that require varying cross-sectional profiles. It is less suitable for complex or intricate shapes and components that require varying cross-sectional profiles. The cost of the required machinery, dies, and tooling can be significant. It is not economical for unit or batch production [22]. Material waste (trimming and cutting are often necessary to achieve the desired component lengths and shapes). Limited thickness control.
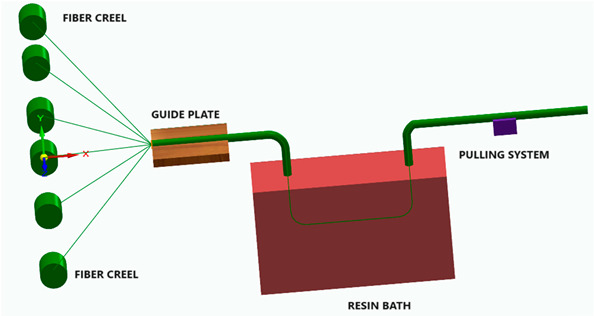 (Adapted form [20]).				
Injection molding	In a typical injection molding process, composite fibers in granules/pellets/beads/powder form are introduced through a hopper, and they are then transported through a heated barrel with a screw. Once the necessary amount of material is melted within the barrel, the screw propels it through a nozzle into the mold, where it cools and is obtained the desired shape [18].	Automotive parts (engine covers, door panels, and lightweight structural components); enclosures for electronic devices and components [18]; medical devices (instrument housings, patient-monitoring equipment, and diagnostic devices).	High precision. Very low cycle times, enabling the rapid production of parts in high volumes, which is especially advantageous for mass production [18]. Reduced material waste.	High initial investment due to the specialized equipment and tooling. Complex tooling. Design limitations. FRP parts may require additional finishing or coating processes to achieve a smooth surface finish.
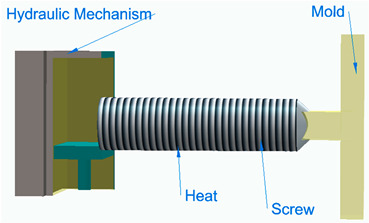 (Adapted form [20]).				
Compression molding	It utilizes metal molds, which are preheated within a temperature range of 250 °F to 400 °F and then mounted onto substantial hydraulic or mechanical molding presses. The compression molding process involves placing a resin charge into a mold. One of the mold parts remains fixed, while the other movable mold part applies heat and pressure to shape the material into the desired structure [20].	The production of high-strength FRP components and high-volume, moderately curved parts across a wide size range (automotive components, aerospace parts, decorative panels, cladding, and architectural elements).	It is a quick and precise method. High productivity. Excellent dimensional stability. The resulting FRP parts feature two exceptionally smooth surfaces and consistent part-to-part quality in comparison to other FRP manufacturing methods like injection molding, resin transfer molding, and vacuum-assisted resin transfer molding. It is one of the most cost-effective molding technologies. It minimizes material waste, making it advantageous for working with high-cost compounds, and it requires minimal post-processing and finishing costs [17].	Limitations in producing complex geometries. High tooling costs. Variability in part quality. High energy consumption. Limited automation for complex parts.Challenges in maintaining precise part thickness control.
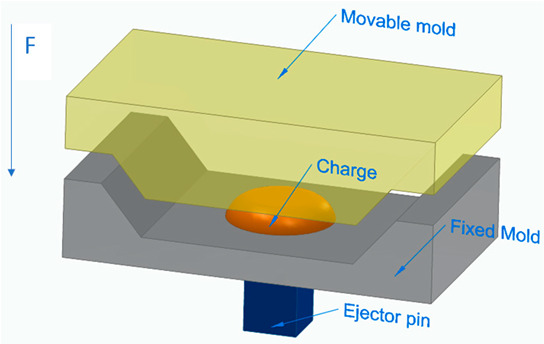 (Adapted form [20]).				
Resin transfer molding	In this process, the initial step involves placing a dry fiber preform within the mold cavity. Subsequently, the mold is sealed, and resin is injected under pressure, facilitated by either vacuum or an injection device until the entire cavity is thoroughly saturated with resin. Finally, the resin-impregnated preform is subjected to curing, cooling, and subsequent demolding.	Truck panels, boat hulls, and aerospace and wind turbine blade products [17].	It allows for the production of components with superior strength and a surface finish that closely replicates the mold’s surface [18]. It allows for flexibility in choosing both the material and its orientation, including the use of 3D reinforcements [20]. Minimum percentage of volatile emissions during processing [17].	High tooling costs. Achieving a mold shape that facilitates uniform resin distribution to all parts of the component demands rigorous testing and the application of advanced fluid dynamic simulations. The relatively slow curing time for the parts and the potential for displacement of reinforcement fibers during the resin transfer process [17].
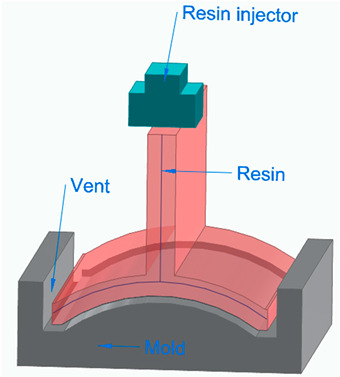 (Adapted form [20]).				
Vacuum-assisted resin transfer molding	The process begins with arranging cloth fabrics or fibers into a preform according to the desired configuration. Typically, these fabrics are held together by a binder and pre-compressed to match the mold’s shape. A second matching mold tool is then secured over the first and vacuum-sealed, functioning as a flexible vacuum bag. The next step involves injecting pressurized resin into the mold cavity, assisted by a vacuum. Once impregnation is complete, the FRP composite part is left to cure at room temperature, with optional post-curing [17].	Aircraft fuselage sections, wind turbine blades, aircraft landing gear doors, large composite panels, and components with minimal void content and a high fiber content [17].	Adaptable tooling design and material options. Ease of mold geometry adjustments. Capacity to create high-quality, load-bearing, and structurally strong complex structures. It achieves remarkable fiber-to-resin ratios, up to 70%, virtually eliminating void. It uses low injection pressures, typically around 1 atm.	Fiber misalignment. Voids. Complex mold design. Since only one side of the part comes into contact with the tool, it restricts the attainment of a single smooth surface. The challenge of managing variations in part thickness.
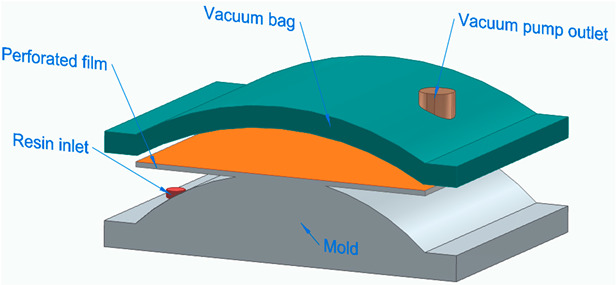 (Adapted form [20]).				
Filament winding	It uses a rotating mandrel as the mold. Continuous reinforcement fibers are pulled from roving and guided through a heated resin bath. These resin-infused continuous fibers are then wound around the rotating mandrel, which is shaped internally according to the desired product design [20].	Cylinders, pipes, bicycles, fuel storage and chemical tanks, stacks, rocket motor cases, pressure vessels, drive shafts, aerospace components, military armaments, power and transmission poles [17].	High strength and stiffness because it uses continuous fibers.High strength-to-weight ratio and product uniformity [20]. It allows for the orientation of the direction of the fibers to obtain optimized characteristics [17]. Reduced labor content.	Limitation in structures with convex shapes. The mandrel is enclosed within the winding, making it challenging to precisely align the fiber along the entire length of the composite and the mandrel. The outer surface of the composite remains unmolded, resulting in an unaesthetic appearance [17].
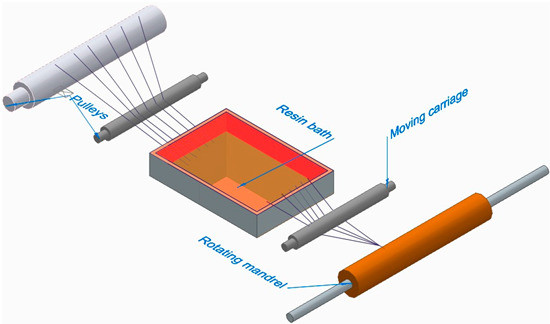 (Adapted form [20]).				
Automated tape lay-up (ATL)	Wide unidirectional tapes are applied to a part of a mold using a roller system equipped with different degrees of articulation, which can vary based on the complexity of the part being produced. Automated tape lay-up essentially imitates the manual process of laying down unidirectional tape but does so at increased speeds [24].	Large, flat, or single-curvature composite structures [18]	Precision. Reduced labor. Improved material efficiency. It is suitable for larger parts. It offers enhanced process control.	The cost associated with acquiring the necessary specialized equipment makes the technology inaccessible for small-to-medium-sized manufacturers [18]. Limitations in terms of part complexity. It requires additional training and expertise for operators.
Automated fiber placement(AFP)	Fiber-reinforced thermoplastic polymer (FRTP) prepreg tapes are applied to a tool, typically using a robotic arm equipped with a fiber placement head. The feeding unit of the fiber placement head directs the prepreg tapes onto the tool. A heat source warms the tape at the nip point, and a compaction roller presses it onto the substrate during the process. The tape is then sliced into strips of predetermined lengths by a cutting unit. The AFP process is managed by a computer program to arrange the FRTP prepreg tapes in the desired lay-up pattern [25].			
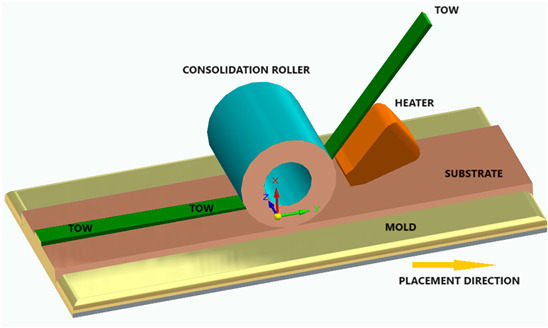 (Adapted form [23]).				
Electrospinning	It is an electrostatic fiber fabrication method that uses electrical forces to create continuous fibers ranging from two nanometers to several micrometers in diameter. This process involves expelling a polymer solution through a spinneret, resulting in the formation of a continuous fiber [18], and the fibers are then collected at the apparatus. As a result, it has promising applications in various fields.	Biomedical sector (wound healing, tissue engineering scaffolds, drug delivery, as a membrane in biosensors, immobilization of enzymes, and cosmetics) [18].	Improved physical and mechanical properties.Flexibility in controlling process parameters. High surface area-to-volume ratio [18]	Limited production rate. Challenges in achieving precise fiber diameters. The equipment and associated materials can be expensive. The use of high voltages in the electrospinning process can pose safety risks.
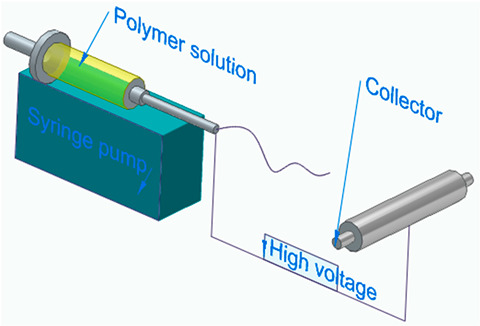 (Adapted form [20]).				
Additive manufacturing (AM)	Additive manufacturing techniques for continuous fiber-reinforced composites encompass methods such as fused deposition modeling (FDM), fused filament fabrication (FFF), directed energy deposition (DED), and laminated object manufacturing (LOM). The most used AM technique is FDM due to some advantages such as simple working principle, low production cost, and efficient and rapid production [23].	It is particularly valuable in industries where lightweight, strong, and complex components are essential.	High level of geometrical complexity. Computer-aided designing eliminates the necessity of molds, decreasing the cost and manufacturing time. Flexibility in choosing both the volume of fibers and their orientation. Reduces material waste material and cycle time [18].	The lack of materials with structural capabilities. The materials presently available generally lack the durability required to serve as a finished product or component. The product size is the print area [26].
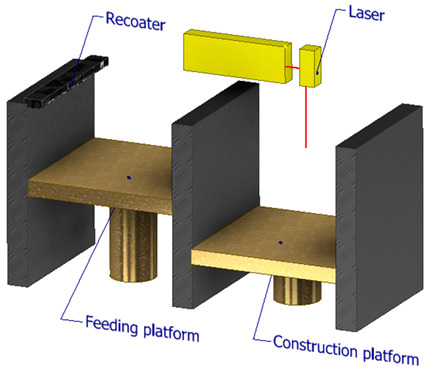 (Adapted form [23]).				

**Table 4:** Mechanical properties of FRP material types.

Property	AFRP	BFRP	CFRP	GFRP
Density (g/cm^3^)	1.25~1.45	1.90~2.10	1.50~2.10	1.25~2.50
Tensile Strength (MPa)	1720~3620	600~1500	600~3920	483~4580
Elongation (%)	1.4~4.4	1.2~2.6	0.5~1.8	1.2~5.0
Young’s Modulus (GPa)	41~175	50~65	37~784	35~86
Coefficient of Linear Expansion (10^–6^/°C)	–6.0~2.0	9.0~12.0	–9.0~0.0	6.0~10.0

**Table 5:** Mechanical properties of high-performance sheet materials.

Type of Sheet		Tensile Strength (MPa)	Elastic Modulus (GPa)	Fracturing Strain (%)
Aramid		200~2500	73~120	1.8~3.0
E-Glass		1500	80	1.9
Carbon	High strength	3400	230	1.5
	High modulus	1900	540	0.35
PBO *		3500	240	1.5
Dyneema		1832	60	3.08
PET *		923	6.7	13.8
PEN *		1028	22.6	4.5
PAF *		1730	40	6

* PBO (Polypara-phenylene-Benzo-bis-Oxazole), PET (Polyethylene Terephthalate/polyester), PEN (Polyethylene Naphthalate), PAF (Polyacetal).

**Table 6:** Typical properties of CFRP [29,30].

Property	Carbon Fiber			
	Pitch Carbon		Polyacrylic Nitril Carbon	
	Common	High Modulus	High Modulus	High Strength
Density (g/cm^3^)	1.6~1.7	1.9~2.1	1.8~2.0	1.7~1.8
Tensile Strength (MPa)	764~980	2940~3430	2450~3920	3430
Young’s Modulus (Pa)	37~39	392~784	343~637	196~235
Elongation (%)	2.1~2.5	0.4~1.5	0.4~0.8	1.3~1.8
Coefficient of Thermal Expansion (10^–6^/°C)	–0.6 up to –0.2	–1.2 up to –0.1	–1.2 up to –0.1	–0.6 up to –0.2

**Table 7:** Typical properties of GFRP [29,30].

Trade Name	E-Glass	S-Glass	C-Glass	AR-Glass
Density (g/cm^3^)	2.5	2.5	2.5	2.27
Tensile Strength (MPa)	3450	4580	3300	1800~3500
Extensionto Break (%)	2.4	3.3	2.3	2.0~3.0
Modulus of Elasticity (GPa)	72.4	85.5	69	70~76
Coefficient of Thermal Expansion (10^–6^/°C)	5.0	2.9	-	-

**Table 8:** Typical properties of AFRP [29,30].

Property	Aramid Fiber					
	Twaron	Twaron HM	Technora H	Kevlar 29	Kevlar 49	Kevlar 149
Density (g/cm^3^)	1.44	1.45	1.39	1.44	1.44	1.44
Tensile strength (MPa)	3000	3000	3000	2760	3620	3450
Modulus of elasticity (GPa)	80	124	70	62	124	175
Extension to break (%)	3.3	2.0	4.4	4.4	2.2	1.4

**Table 9:** Properties of thermoset resins for FRP matrices [29].

Property	Resin		
	Epoxy	Vinylester	Polyesters
Density (g/cm^3^)	1.2~1.4	1.15~1.35	1.1~1.4
Tensile Strength (MPa)	55~130	73~81	34.5~104
Young’s Modulus (GPa)	2.75~4.10	3.0~3.5	2.1~3.45
Poisson’s Ratio	0.38~0.40	0.36~0.39	0.35~0.39
Saturation (%)	0.08~0.15	0.14~1.3	0.15~0.60
Coefficient of Thermal Expansion (10^–6^/°C)	45~65	50~75	55~100

**Table 10:** Characteristics of GRP pipes.

Description		RTRP	AWWAM45
Diameter (mm)		2400	-
Thickness (mm)		44.5	-
Hoop	Tensile strength (MPa)	303.3	14~550
	Tensile modulus of elasticity (GPa)	41.6	3.5~34.5
	Bending modulus of elasticity (GPa)	22.3	-
Axial	Tensile strength (MPa)	164.0	14~550
	Tensile modulus of elasticity (GPa)	12.4	3.5~34.5
	Compressive strength (MPa)	211.0	69~275
	Compressive modulus of elasticity (GPa)	12.2	-
	Bending strength (MPa)	175.0	28~480
	Bending modulus of elasticity (GPa)	11.0	6.9~34.5
Izod impact strength (J/m)		256.0	-
Coefficient of linear expansion (1/°C)		1.6 × 10^−5^	1.4 × 10^−5^~5.4 × 10^−5^
HDB (forstress) (MPa)		97.5	-
HDB (forstrain) (%)		0.653	-
Poisson’sratio		0.159	-

**Table 11:** FRP mechanical characteristics according to Wavistrong Engineering [105].

Property	Symbol	Test Method	Winding Angle (ω)			Unit
			55°	63°	73°	
Axial tensile stress	E_x_	ASTM D 2105	65	55	40	MPa
Axial tensile modulus		ASTM D 2105	10,500	10,000	10,000	MPa
Hoop tensile stress		ASTM D 2290	210	260	400	MPa
Hoop tensile modulus		ASTM D 2290	20,500	27,500	37,000	MPa
Shear modulus	E_s_		11,500	9500	7000	MPa
Axial bending stress	E_x_	ASTM D 2925	80	65	50	MPa
Axial bending modulus			10,500	10,000	10,000	MPa
Hoop bending stress	E_H_	ASTM D 2412	90	120	160	MPa
Hoop bending modulus		ASTM D 2412	20,500	27,500	37,000	MPa
Poisson ratio axial/hoop	N_XY_		0.65	0.62	0.47	-
Poisson ratio hoop/axial	N_YX_		0.38	0.26	0.15	-

**Table 12:** FRP mechanical characteristics according to Fiberbond [105].

Density (g/cm^3^)	1.7
Shear Modulus (GPa)	6.9
Coefficient of Linear Expansion (1/°C)	1.6 × 10^−5^
Thermal Conductivity (W/(m·°C))	0.0019
Minor Poisson’s Ratio (ν_min_ = ν_ha_)	0.55
Major Poisson’s Ratio (E_a_/E_h_·ν_ah_ = ν_ah_)	0.35
Hazen Williams Coefficient	150

**Table 13:** The calculation expressions for wall thickness.

$t=\frac{PD}{2SE}$ $t=\frac{PD}{2\left(SE+PY\right)}$ $t=\frac{D}{2}(1-\sqrt{\frac{SE-P}{SE+P}}$) $t=\frac{P\left(d+2c\right)}{2\left(SE-P\left(1-Y\right)\right)}$	$t=\frac{PD}{2S+P}$ $t=\frac{PD}{2SF+P}$
Metallic materials	FRP pipes(nonmetallic)

*t*—pressure design thickness (reinforced only); *P*—internal design gage pressure; *D*—outside diameter of pipe; *S*—stress value for material from Table A-1 [101]; *E*—quality factor from Table A-1A or A-1B [101] (usually ranges from 0.8 to 1.0); *Y*—coefficient from Table 304.1.1 [101] (0.4 for most metallic materials below 482 °C); *c*—sum of the mechanical allowances plus corrosion and erosion allowances; F—service (design) factor (usually <1.0 when using cycle HDBS and <0.5 when using static HDBS) (HDBS = LTHP from D2992).

**Table 14:** The calculation expressions for wall thickness, according to [106,107].

$t=\frac{P\cdot D_{i}}{\frac{2{\cdot S}_{h}}{F}}$	$t=\frac{P{\cdot D}_{i}}{2\cdot \left(0.001\cdot E_{h}\right)}$
Contact molded(laminated)	Filament wound

*t*—total wall thickness; *P*—total internal pressure; *D_i_*—inside diameter; *S_h_*—ultimate hoop tensile strength (since this equation is for contact molded construction, this property is obtained from a flat plate test according to [107]); *F*—design factor = 10.0; *E_h_*—hoop tensile modulus.

**Table 15:** The calculation expressions, according to [100,106,107].

ASME RTP-1 3A-310 [24]	ISO 14692 [18]
$P_{a}=\frac{K\cdot \frac{E_{r}}{F}\cdot \frac{D_{o}}{L}\cdot {\left(\frac{t}{D_{o}}\right)}^{25}}{1-0.45\cdot {\left(\frac{t}{D_{o}}\right)}^{0.5}}$ $E_{r}=\sqrt{E_{a}\cdot E_{h}}$ $K=4-0.75\cdot \frac{E_{r}}{1000000}$	$P_{c}=20E_{h}{\left(\frac{t}{D}\right)}^{3}$ $P_{allowable}=\frac{P_{c}}{3}$

*P_a_*—allowable external pressure; *E_a_*—axial tensile modulus; *E_h_*—hoop tensile modulus; *F*—design factor = 5.0; *D_o_*—outside diameter; *L*—design length of vessel section (if this were applied to piping, it would normally be the distance between stiffener rings, if used, or between hangers, with 360° contact, or between secondary overlays and other external stiffeners); *t*—nominal wall thickness; *P_c_*—buckling collapse pressure (bar); *E_h_*—hoop modulus (MPa); *E_r_*—resultant modulus(MPa); *D*—mean pipe diameter (mm); *P_allowable_*—allowable external pressure (bar).

**Table 16:** The calculation expressions, according to [106,107,109].

BS 7159:1989	ASME Boiler and Pressure Vessel Code, Section X
$p_{e}=\frac{20\cdot E_{lam}({\frac{t_{d}}{D_{i}+2\cdot t_{d}})}^{30}}{F_{s}}$	$P_{a}=\frac{K\cdot \frac{E_{r}}{F}\cdot {\left(\frac{t}{D_{o}}\right)}^{25}}{1-0.45\cdot {\left(\frac{t}{D_{o}}\right)}^{0.5}};if\;L_{c}>L$ $E_{r}$ = $\sqrt{E_{1}\cdot E_{2}}$ $K=3.6-\frac{2\cdot E_{r}}{E_{1}+E_{2}}$ = $L_{c}=1.14\cdot {\left(1-v_{1}\cdot v_{2}\right)}^{0.25}\cdot D_{o}\cdot {\left(\frac{D_{o}}{t}\right)}^{0.5}$

*P_a_*—allowable external pressure; *E*_1_—axial tensile modulus; *E*_2_—hoop tensile modulus; *E_r_*—resultant modulus; *F*—design factor = 5.0; *D_o_*—outside diameter; *L*—design length of vessel section (if this were applied to piping, it would normally be the distance between stiffener rings, if used, or between hangers, with 360° contact, or between secondary overlays and other external stiffeners); *t*—shell structural thickness (0.25 in. minimum); *L_c_*—critical length; *P_e_*—allowable external pressure (bar); *E_lam_*—modulus of elasticity of the laminate (MPa); *t_d_*—design thickness of the reference laminate (mm); *D_i_*—internal diameter (mm); *F_s_*—factor of safety = 4.0.

## Data Availability

Data are contained within the article.
